# Discovery of Rezatapopt (PC14586), a First-in-Class, Small-Molecule Reactivator of p53 Y220C Mutant in Development

**DOI:** 10.1021/acsmedchemlett.4c00379

**Published:** 2024-11-04

**Authors:** Binh T. Vu, Romyr Dominique, Bruce J. Fahr, Hongju H. Li, David C. Fry, Lizhong Xu, Hong Yang, Anna Puzio-Kuter, Andrew Good, Binbin Liu, Kuo-Sen Huang, Naoko Tanaka, Thomas W. Davis, Melissa L. Dumble

**Affiliations:** 1 Discovery Chemistry, PMV Pharmaceuticals, Inc., 400 Alexander Park Drive, Suite 301, Princeton, New Jersey 08540, United States; 2 The Chemistry Research Solution, 360 George Patterson Blvd., Suite 108, Bristol, Pennsylvania 19007, United States; 3 Discovery Biology, PMV Pharmaceuticals, Inc., 400 Alexander Park Drive, Suite 301, Princeton, New Jersey 08540, United States; 4 CAnDiD Consulting, 52 High Hill Road, Wallingford, Connecticut 06492, United States; 5 WuXi AppTec (Tianjin) Co., 168 Nanhai Road, Tianjin 300457, China; 6 Cepter Biopartners, 123 Metro Boulevard, Nutley, New Jersey 07110, United States

**Keywords:** Rezatapopt, p53, Y220C, reactivator, solid tumor, mutation

## Abstract

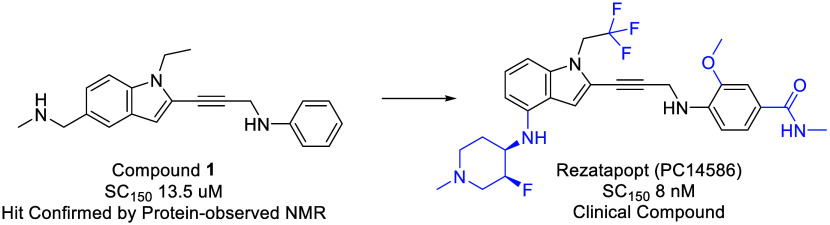

p53 is a potent transcription factor that is crucial in regulating cellular responses to stress. Mutations in the *TP53* gene are found in >50% of human cancers, predominantly occurring in the DNA-binding domain (amino acids 94–292). The Y220C mutation accounts for 1.8% of all of the *TP53* mutations and produces a thermally unstable protein. Rezatapopt (also known as PC14586) is the first small-molecule p53 Y220C reactivator being evaluated in clinical trials. Rezatapopt was specifically designed to tightly bind to a pocket created by the *TP53* Y220C mutation. By stabilization of the p53 protein structure, rezatapopt restores p53 tumor suppressor functions. In mouse models with established human tumor xenografts harboring the *TP53* Y220C mutation, rezatapopt demonstrated tumor inhibition and regression at well-tolerated doses. In Phase 1 clinical trials, rezatapopt demonstrated a favorable safety profile within the efficacious dose range and showed single-agent efficacy in heavily pretreated patients with various *TP53* Y220C mutant solid tumors.

The tumor suppressor protein p53 is a critical transcription factor that regulates cellular responses to stress.^1,2^ However, cancer cells with mutations in the *TP53* gene can evade p53 tumor-suppressive effects, promoting cancer cell growth and tumorigenesis. More than 50% of human cancers harbor mutations in the *TP53* gene that encodes the p53 protein, and most of these are missense mutations occurring in the DNA binding domain of p53 (amino acids 94–292). Among the various *TP53* mutations observed across numerous tumor types, the Y220C mutation is the ninth most frequent, accounting for 1.8% of all p53 mutations.^3^ The amino acid substitution creates a pocket in the p53 Y220C mutant protein that renders it structurally unstable at physiological temperatures. Targeting the p53 Y220C mutant protein using a small molecule has been proposed as a potential therapeutic strategy for anticancer treatment.^4^

Rezatapopt (also known as PC14586) is a small molecule reactivator of the p53 Y220C mutant and is being evaluated in clinical trials. It was designed to fit tightly into the pocket within the p53 Y220C mutant protein, thereby stabilizing the p53 protein structure in the wild type (WT) conformation and restoring p53 tumor suppressor functions. The preclinical characterization of rezatapopt has recently been disclosed.^5^ This account describes the chemistry efforts that led to the discovery of rezatapopt.

At the outset of our hit generation campaign, several small molecules targeting the p53 Y220C mutant protein were identified. Two notable examples, supported by crystallographic data, were the compounds from the carbazole series^6^ (PhiKan83) and the iodophenol series^7^ (PK1596) (Figure 1). To assess the *in vitro* potency of the p53 Y220C reactivators, a time-resolved fluorescence resonance energy transfer (FRET) assay was developed. In this assay, recombinant His-tag Y220C p53 DNA binding domain binds to biotin-labeled consensus DNA, and the FRET signal is measured between the allophycocyanin-conjugated anti-His-tag antibody and europium-conjugated streptavidin. The substrate concentration required to increase DNA binding by 1.5-fold (SC_150_) could then be determined. The carbazole compound (PhiKan83) enhanced DNA binding with an SC_150_ of 37.2 μM. The iodophenol compound (PK1596) exhibited greater potency, with an SC_150_ of 1.6 μM.

**Figure 1:**
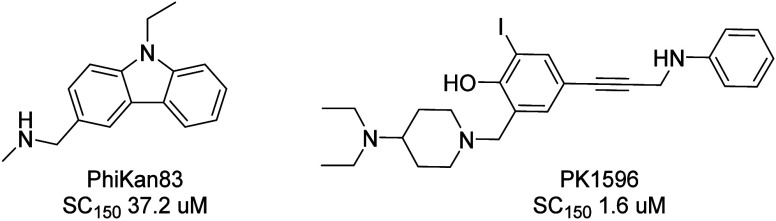
Early p53 Y220C mutant reactivator compounds.

Using structure-based design tools, multiple scaffolds were generated, combining key features of PhiKan83 and PK1596 compounds. Compound **1** emerged as the first starting point for lead generation (Figure 2). The indole scaffold was selected due to its synthetic flexibility, allowing for the introduction of diverse functional groups (Figure 3). Indole is widely acknowledged as a “privileged structure” in drug discovery, given its presence in numerous natural products and bioactive molecules.^8^ The indole scaffold was designed with a hydrophobic anchor (R_3_) and an acetylene linker to enable targeting of the adjacent subsite. Since R_1_ and R_2_ groups projected toward the solvent, a range of polar groups were incorporated to investigate the effects on binding and drug metabolism and pharmacokinetic (DMPK) properties. To avoid following false positives, the binding activity of potential hit compounds was confirmed by protein-observed nuclear magnetic resonance spectroscopy, comparing ^1^H/^15^N-heteronuclear single quantum coherence spectra of uniformly ^15^N-labeled Y220C mutant p53 DNA binding domains with and without the hit compounds.

**Figure 2:**
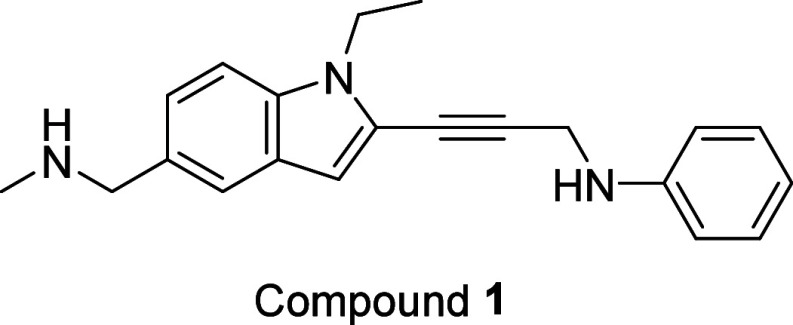
Structure of compound **1**.

**Figure 3:**
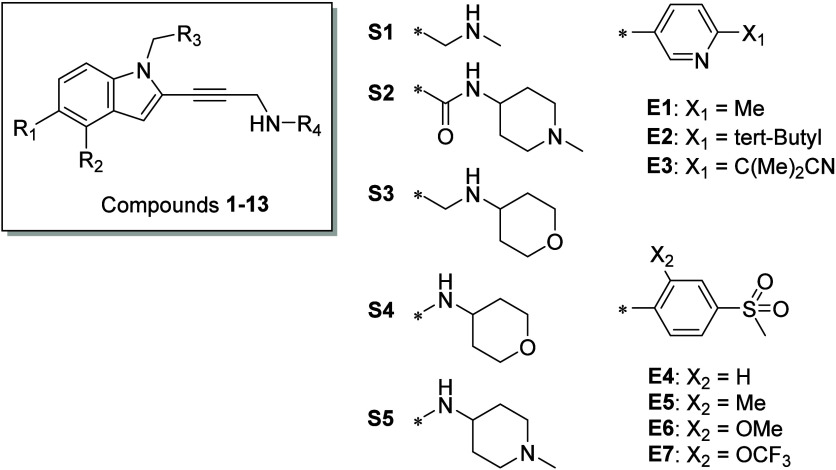
Scaffold of compounds **1–13** and the various substituents assessed. Please refer to Table 1 for specific R groups.

The synthesis of these compounds is exemplified by compound **13** (Figure 4). Detailed experimental procedures for the synthesis of compound **13** and structural information and characterization data for all compounds can be found in the Supporting Information. A convergent synthesis utilized the Sonogashira coupling reaction of the 2-iodo indole **16** and the alkyne **18** to produce compound **13**. The iodo indole **16** was prepared from the iodo aniline **15**, and the alkyne **18** was prepared from the aniline **17**, using standard synthetic methods.

**Figure 4:**
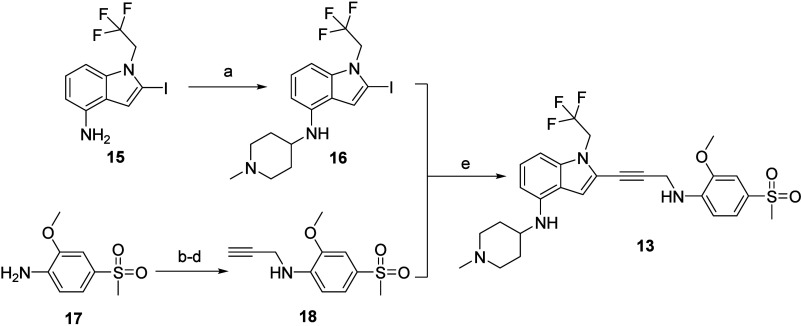
Synthesis of compound **13**. Reagents and conditions: (a) SnCl_2_, NaBH_3_CN, 1-methyl-4-piperidone; (b) Boc_2_O, dioxane; (c) NaH, DMF, propargyl bromide; (d) EtOAc/HCl; (e) CuI, i-Pr_2_NH, Pd(PPh_3_)_4_, DMSO.

Compound **1** emerged as our validated hit, exhibiting an SC_150_ of 13.6 μM, and therefore, the hit expansion process centered on compound **1**. Representative compounds and their biological data are shown in Table 1.

**Table 1:** Biological Data of Early Compounds **1–13**

**Compound**	**R**_**1**_	**R**_**2**_	**R**_**3**_	**R**_**4**_	**SC**_**150**_^a^**(μM)**
**1**	S1	H	CH_3_	C_6_H_5_	13.6
**2**	S2	H	CH_3_	C_6_H_5_	>150
**3**	S3	H	CH_3_	C_6_H_5_	26.5
**4**	S3	H	CH_3_	E1	6.3
**5**	S1	H	CH_3_	E2	6.4
**6**	S3	H	CH_3_	E3	1.3
**7**	S3	H	CF_3_	E3	0.48
**8**	H	S3	CF_3_	E3	1.300
**9**	H	S4	CF_3_	E3	0.105
**10**	H	S5	CF_3_	E3	0.054
**11**	H	S5	CF_3_	E4	0.027
**12**	H	S5	CF_3_	E5	0.030
**13**	H	S5	CF_3_	E6	0.023
**14**	H	S5	CF_3_	E7	0.030

a SC_150_, substrate concentration required to increase DNA binding by 1.5-fold.

The hit expansion process initially focused on the 5-substituted indole compounds (R_2_ = H, compounds **1**–**7**), but they did not reach adequate potency (with SC_150_ values of >100 nM) and were metabolically unstable. For example, compound **1** had a half-life (*T*_1/2_) of 4 min in human liver microsomes. However, there was a significant improvement in potency with fluorine substitution at the hydrophobic anchor (R_3_ = CF_3_, compound 7). The trifluoromethyl group had a favorable interaction with the sulfur atom of Cysteine-220, also reported by Fersht et al. for the carbazole series.^9^ The trifluoromethyl group remained the best anchor throughout the hit expansion and drug discovery campaign. Assessment of the 4-substituted indole compounds (R_1_ = H, compounds **8**–**14**) led to a breakthrough regarding the potency of the compounds. Our molecular modeling predicted that the side chain of Threonine-150 within the p53 Y220C protein would form a hydrogen bond with the ligand. This interaction helped to orient the ligand in the binding pocket of the p53 Y220C mutant protein and enhance other interactions such as hydrophobic and van der Waals interactions. The optimal position of the nitrogen atom was determined in compound **9**, whereas adding a methylene group between the nitrogen atom and the indole scaffold resulted in a significant loss of potency, as shown by compound **8**. With the nitrogen atom in place, various polar groups were assessed, and it was determined that basic nitrogen, such as that in the piperidine ring, was preferred for maintaining the potency of the compound (R_2_ = S5). Many substituents can be tolerated for the aromatic ring occupying the subsite. Substituents such as methoxy (E6) or trifluoromethoxy (E7) group at the ortho position of the aniline ring did not significantly impact the potency of the compound but provided improved pharmacokinetic (PK) exposure (Figure 3).

As the lead compounds gained potency, with SC_150_ values reaching below the 100 nM threshold, X-ray crystallization studies confirmed the binding mode of the lead compounds. The crystal structure of compound **10** complexed with the p53 Y220C mutant protein was successfully determined at 1.70 Å resolution. The p53 Y220C mutant protein used for X-ray crystallization trials consists of the DNA binding domain spanning amino acids 94–312, which was stabilized by four additional mutations (M133L, V203A, N239Y, N268D).^4,10^ The X-ray structure confirmed that compound **10** binds the p53 Y220C mutant protein within a central cavity that is created by the tyrosine-to-cysteine substitution in the p53 Y220C mutant protein, with the indole moiety occupying this space and the trifluoromethyl group extending deeply into the hydrophobic pocket. The 4-aminopiperidine ring extends toward the solvent, and the alkyne linker allows the aromatic ring to reach the adjacent subsite. This enables a favorable CH-π stacking interaction of the aromatic ring with Proline-153. Two key hydrogen bonds between the p53 Y220C mutant protein and compound **10** were observed: one interaction with the carbonyl of Cysteine-220 and another with the side chain of Threonine-150 (Figure 5).

**Figure 5:**
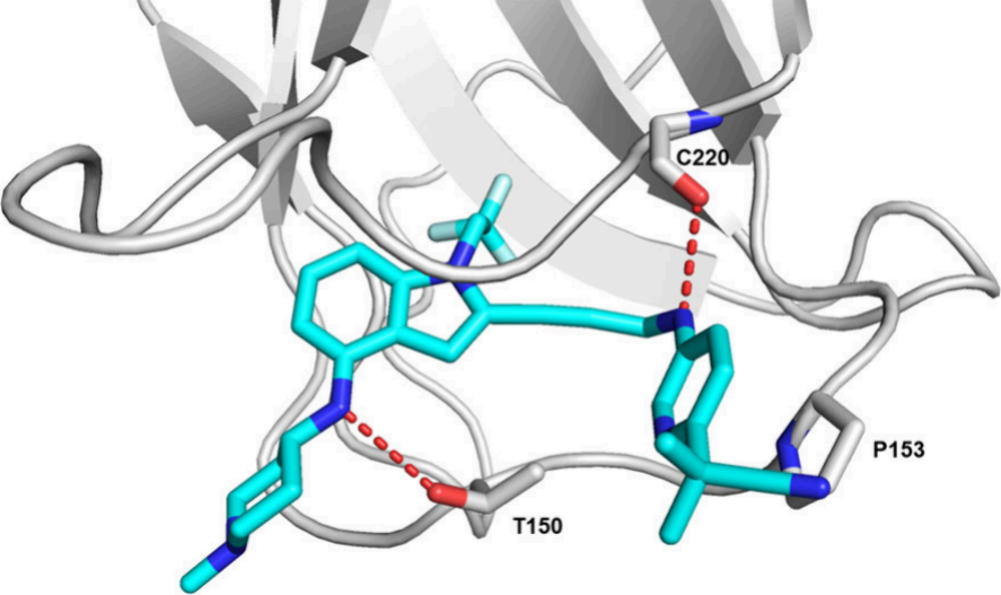
Crystal structure of the p53 Y220C mutant protein bound to compound **10**. The p53 Y220C mutant protein is colored gray, with key residues shown as colored stick models (nitrogen atoms colored blue and oxygen atoms highlighted in red). Compound **10** is shown in color, where carbon atoms are cyan, nitrogen atoms are blue, and fluorine atoms are light blue. The dashed red lines indicate hydrogen bonds between the p53 Y220C mutant protein and compound **10**. PDB code: 9BR4.

We also evaluated the effects of these compounds on the growth and viability of four cancer cell lines *in vitro*. Two of the cell lines (NUGC-3, gastric cancer cell line with a *TP53* Y220C mutation and T3M-4, pancreatic carcinoma cell line) have the *TP53* Y220C mutation leading to the p53 Y220C mutant protein; SJSA-1 is a p53 WT cell line, and NUGC-3-KO is an isogenic p53 knockout cell line.^11^ After incubating the cells with the compounds for five days, cell viability was measured using the 3-(4,5-dimethylthiazol-2-yl)-2,5-diphenyltetrazolium bromide (MTT) assay.

Significant efforts were dedicated to the optimization of compound **13**, and a successful approach involved fluorine substitution of the piperidine ring. Fluorine substitution often plays a crucial role in medicinal chemistry, exerting a substantial impact on the characteristics of drug molecules.^12,13^ Incorporation of fluorine atoms can yield diverse effects and improvements in a drug’s pharmacological properties. In the case of the piperidine ring in compound **13**, several key effects of fluorine substitution were predicted, including enhanced hydrogen bonding with Threonine-150, increased conformational rigidity of the piperidine ring, and reduced basicity. The substitution of the piperidine ring in compound **13** resulted in two pairs of stereoisomers. Among these, the *cis* racemic **20** exhibited over 3-fold higher potency in the MTT assay. By separating the two enantiomers of compound **20** using chiral supercritical fluid chromatography, we obtained compound **22**, which demonstrated approximately 7-fold increased potency compared with compound **13** (Figure 6; Table 2). Compound **22** is significantly more potent than its enantiomer, compound **21**. We postulated that the fluorine atom on the piperidine ring of compound **22**, which preferentially stays in the axial position, can form a better hydrogen bond with the N-H group of Threonine-150. The enhanced cellular potency of compound **22** can be attributed to the enhanced permeability. Specifically, compound **13** and compound **22** exhibited Caco-2 P_app_ values of 11.8 × 10^–6^ cm/s and 19.9 × 10^–6^ cm/s, respectively. Recently, insights into the conformational behavior of *cis-*fluorinated piperidines were reported.^14^

**Figure 6:**
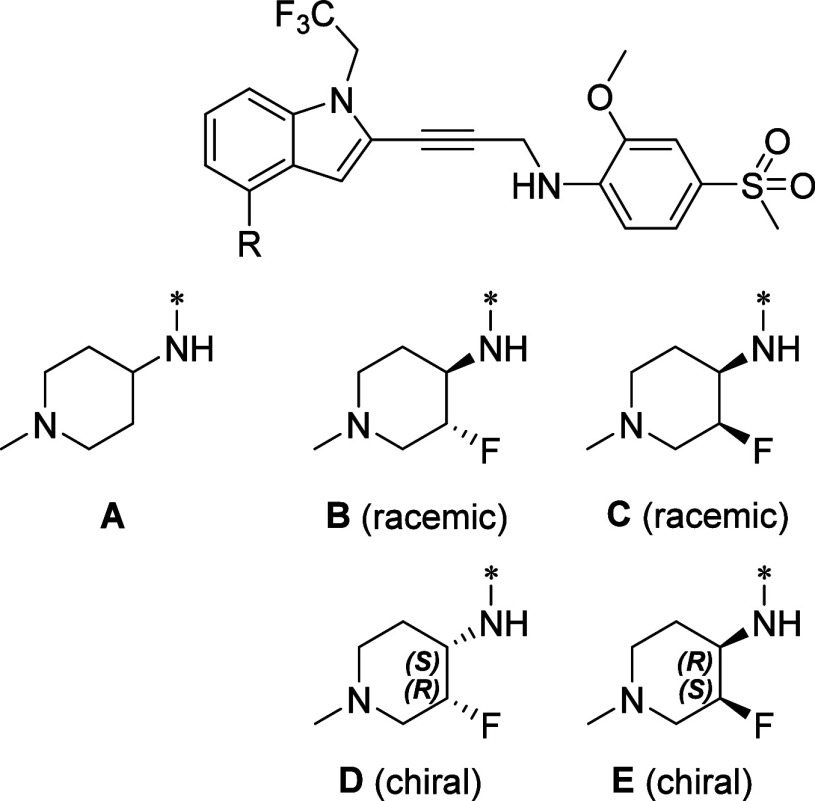
Compound **13** and analogues resulting from the substitution of the piperidine ring.

**Table 2:** Biological Data of Fluoro-Substituted Compounds **19–22** Compared with Compound **13**^a^

				**Mouse PK Exposure (PO, 50 mg/kg)**	
**Compound**	**R**	**SC**_**150**_**(nM)**	**NUGC-3 MTT IC**_**50**_**(μM)**	***C***_**max**_ (ng/mL)	**AUC**_**0-last**_**(ng·h/mL)**
**13**	A	23	2.1	903	3545
**19**	B	20	3.0	nd	nd
**20**	C	9	0.7	nd	nd
**21**	D	20	2.3	2353	23233
**22**	E	8	0.3	2103	12567

a AUC_0-last_, area under the curve from dosing to the time of the last measured concentration; *C*_max_, maximum drug concentration; IC_50_, half-maximal inhibitory concentration; MTT, 3-(4,5-dimethylthiazol-2-yl)-2,5-diphenyltetrazolium bromide; nd, not determined; NUGC-3, gastric cancer cell line with a *TP53* Y220C mutation; PK, pharmacokinetic; PO, by mouth; R, fluorine substitution of piperidine ring as shown in Figure 6; SC_150_, substrate concentration required to increase DNA binding by 1.5-fold.

After discovering the fluorine-substituted piperidine, our focus shifted to further optimizing compound **22** by investigating various substituents in the R group (Figure 7). Since R groups extend toward the solvent, we introduced a variety of polar groups to assess their effects on binding affinity and DMPK properties. Table 3 summarizes the biological data and PK exposures of the representative compounds in this series.

**Figure 7:**
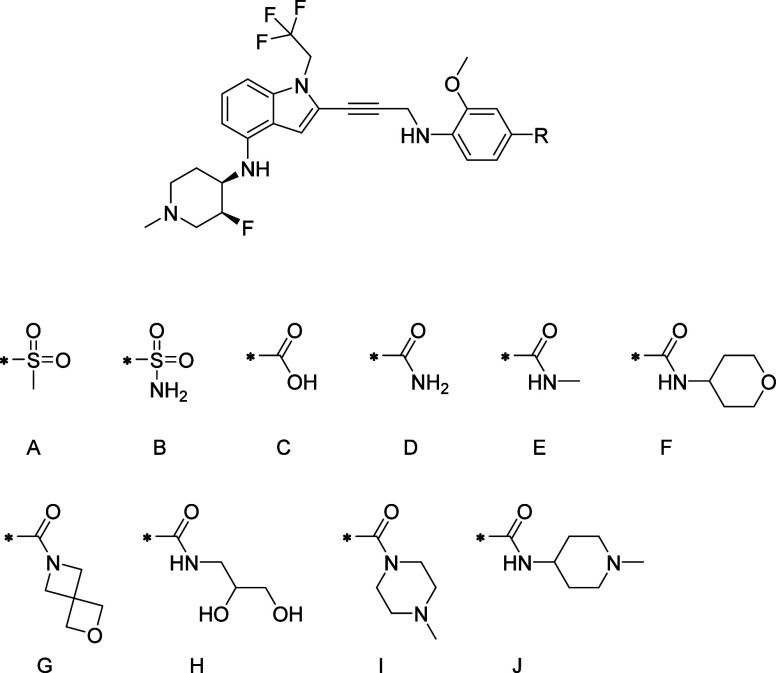
Fluorine-substituted piperidine analogues **22–31**.

**Table 3:** p53 Y220C Mutant Activities and Mouse PK Parameters for Selected Compounds **22–31**^a^

				**Mouse PK Exposure (PO, 50 mg/kg)**	
**Compound**	**R**	**SC**_**150**_**(nM)**	**NUGC-3 MTT IC**_**50**_**(μM)**	***C***_**max**_ (ng/mL)	**AUC**_**0-last**_ (ng·h/mL)
**22**	A	8	0.325	2103	12567
**23**	B	9	0.261	1310	8066
**24**	C	11	0.99	27700	57882
**25**	D	8	0.446	4473	39774
**26**	E	9	0.504	16600	163342
**27**	F	7	0.531	807	3261
**28**	G	7	0.457	2747	15328
**29**	H	8	0.574	54	117
**30**	I	9	0.791	nd	nd
**31**	J	5	1.164	nd	nd

a AUC_0-last_, area under the curve from dosing to the time of the last measured concentration; *C*_max_, maximum drug concentration; IC_50_, half-maximal inhibitory concentration; MTT, 3-(4,5-dimethylthiazol-2-yl)-2,5-diphenyltetrazolium bromide; nd, not determined; NUGC-3, gastric cancer cell line with a *TP53* Y220C mutation; PK, pharmacokinetic; PO, by mouth; R, fluorine substitution of piperidine ring as shown in Figure 7; SC_150_, substrate concentration required to increase DNA binding by 1.5-fold.

Compound **26** (rezatapopt; PC14586), which had the most favorable plasma exposure, was evaluated for its ability to inhibit the growth of NUGC-3 tumor xenografts in nude mice. Oral administration of daily doses of 25 and 50 mg/kg of compound **26** resulted in 33% and 71% inhibition of tumor growth, respectively. Moreover, a higher dose of 100 mg/kg demonstrated an impressive 80% tumor regression.^5,11^ As a comparison, compound **24**, with lower PK exposure and less potency than compound **26**, achieved 54% tumor growth inhibition at a higher dose of 150 mg/kg in the same xenograft model. With a favorable safety profile confirmed in subsequent toxicological studies, compound **26** was selected for further evaluation in human clinical trials.

In conclusion, the combination of structure-based drug design efforts and the incorporation of fluorine substitution has led to the discovery of rezatapopt, the first investigational selective p53 Y220C reactivator. In Phase 1 clinical trials, rezatapopt demonstrated a favorable safety profile in the efficacious dose range, and single-agent clinical efficacy was achieved in heavily pretreated patients across multiple solid tumor types harboring the *TP53* Y220C mutation.^15^ The Phase 2 portion of the PYNNACLE trial will serve as a registrational study and is currently enrolling. This study will further assess the efficacy and safety of rezatapopt as a monotherapy in patients with locally advanced or metastatic solid tumors who have a *TP53* Y220C mutation and are *KRAS* WT. Results from the clinical trial will be reported in subsequent publications.^16^

## Supporting Information Available

Experimental procedures for the synthesis of Compound 13 and Compound 26, characterization data for all compounds, and further details about the TR-FRET, X-ray crystallography, cell proliferation, and in vivo efficacy assays (PDF)

## Abbreviations

DMF: dimethylformamide
DMPK: drug metabolism and pharmacokinetics
DMSO: dimethylsulfoxide
FRET: fluorescence resonance energy transfer
MTT: 3-(4,5-dimethylthiazol-2-yl)-2,5-diphenyltetrazolium bromide
NUGC-3: gastric cancer cell line with a TP53 Y220C mutation
PK: pharmacokinetic
SC_150_: substrate concentration required to increase DNA binding by 1.5-fold
T_1/2_: half-life
T3M-4: pancreatic carcinoma cell line
WT: wild type
