# Supplementary material for: Discovery of Rezatapopt (PC14586), a First-in-Class, Small-Molecule Reactivator of p53 Y220C Mutant in Development
Source: ACS Med Chem Lett. 2024 Nov 4;16(1):34–9. doi: 10.1021/acsmedchemlett.4c00379 (PMC11726359; doi:10.1021/acsmedchemlett.4c00379)
Supplement: Supplementary file 1 — ml4c00379_si_001.pdf [file ml4c00379_si_001.pdf]

SUPPORTING INFORMATION

Discovery of Rezatapopt (PC14586), a First-in-Class, Small-Molecule Reactivator of p53 Y220C Mutant in Development

Authors: Binh T. Vu,[1]* Romyr Dominique,[1] Bruce J. Fahr,[1] Hongju H. Li,[1] David C. Fry,[2] Lizhong Xu,[3] Hong Yang,[3] Anna Puzio-Kuter,[3] Andrew Good,[4] Binbin Liu,[5] Kuo-Sen Huang,[6] Naoko Tanaka,[6] Thomas W. Davis,[3] Melissa L. Dumble[3]

Affiliations: [1]Discovery Chemistry, PMV Pharmaceuticals, Inc., 400 Alexander Park Drive, Suite 301, Princeton, NJ 08540, USA; [2]The Chemistry Research Solution, 360 George Patterson Blvd., Suite 108, Bristol, PA 19007, USA; [3]Discovery Biology, PMV Pharmaceuticals, Inc., 400 Alexander Park Drive, Suite 301, Princeton, NJ 08540, USA; [4]CAnDiD Consulting, 52 High Hill Road, Wallingford, CT 06492, USA; [5]WuXi AppTec (Tianjin) Co., 168 Nanhai Road, Tianjin 300457, China; [6]Cepter Biopartners, 123 Metro Boulevard, Nutley, NJ 07110, USA.

Corresponding Author: Binh T. Vu

Address: PMV Pharmaceuticals, Inc., 400 Alexander Park Drive, Suite 301, Princeton, New Jersey 08540, USA.
Email: bvu@pmvpharma.com

Table of Contents

General Information 4

Safety Statement 4

Preparation of Compound 13 4

Preparation of Compound 26[a] 7

List of compounds and structures 11

Compound 1: N-[3-[1-ethyl-5-(methylaminomethyl)indol-2-yl]prop-2-ynyl]aniline 11

Compound 2: 2-(3-anilinoprop-1-ynyl)-1-ethyl-N-(1-methyl-4-piperidyl)indole-5-carboxamide 11

Compound 3: N-((1-ethyl-2-(3-(phenylamino)prop-1-yn-1-yl)-1H-indol-5-yl)methyl)tetrahydro-2H-pyran-4-amine 11

Compound 4: N-(3-(1-ethyl-5-(((tetrahydro-2H-pyran-4-yl)amino)methyl)-1H-indol-2-yl)prop-2-yn-1-yl)-6-methylpyridin-3-amine 12

Compound 5: 6-(tert-butyl)-3-(3-{1-ethyl-5-[(methylamino)methyl]-2-indolyl}-2-propynylamino)pyridine 12

Compound 6: 2-(5-((3-(1-ethyl-5-(((tetrahydro-2H-pyran-4-yl)amino)methyl)-1H-indol-2-yl)prop-2-yn-1-yl)amino)pyridin-2-yl)-2-methylpropanenitrile 12

Compound 7: 2-methyl-2-(5-((3-(5-(((tetrahydro-2H-pyran-4-yl)amino)methyl)-1-(2,2,2-trifluoroethyl)-1H-indol-2-yl)prop-2-yn-1-yl)amino)pyridin-2-yl)propanenitrile 13

Compound 8: 2-methyl-2-(5-((3-(4-(((tetrahydro-2H-pyran-4-yl)amino)methyl)-1-(2,2,2-trifluoroethyl)-1H-indol-2-yl)prop-2-yn-1-yl)amino)pyridin-2-yl)propanenitrile 13

Compound 9: 2-methyl-2-(5-((3-(4-((tetrahydro-2H-pyran-4-yl)amino)-1-(2,2,2-trifluoroethyl)-1H-indol-2-yl)prop-2-yn-1-yl)amino)pyridin-2-yl)propanenitrile 14

Compound 10: 2-methyl-2-(5-((3-(4-((1-methylpiperidin-4-yl)amino)-1-(2,2,2-trifluoroethyl)-1H-indol-2-yl)prop-2-yn-1-yl)amino)pyridin-2-yl)propanenitrile 14

Compound 11: N-(1-methylpiperidin-4-yl)-2-(3-((4-(methylsulfonyl)phenyl)amino)prop-1-yn-1-yl)-1-(2,2,2-trifluoroethyl)-1H-indol-4-amine 14

Compound 12: 2-[3-(2-methyl-4-methylsulfonyl-anilino)prop-1-ynyl]-N-(1-methyl-4-piperidyl)-1-(2,2,2-trifluoroethyl)indol-4-amine 15

Compound 14: N-(1-methyl-4-piperidyl)-2-[3-[4-methylsulfonyl-2-(trifluoromethoxy)anilino]prop-1-ynyl]-1-(2,2,2-trifluoroethyl)indol-4-amine 15

Compound 19: *Rac*-N-[(3R,4R)-3-fluoro-1-methyl-4-piperidyl]-2-[3-(2-methoxy-4-methylsulfonyl-anilino)prop-1-ynyl]-1-(2,2,2-trifluoroethyl)indol-4-amine 16

Compound 20: *Rac*-N-[(3S,4R)-3-fluoro-1-methyl-4-piperidyl]-2-[3-(2-methoxy-4-methylsulfonyl-anilino)prop-1-ynyl]-1-(2,2,2-trifluoroethyl)indol-4-amine 16

Compound 21: N-[(3R,4S)-3-fluoro-1-methyl-4-piperidyl]-2-[3-(2-methoxy-4-methylsulfonyl-anilino)prop-1-ynyl]-1-(2,2,2-trifluoroethyl)indol-4-amine 17

Compound 22: N-[(3S,4R)-3-fluoro-1-methyl-4-piperidyl]-2-[3-(2-methoxy-4-methylsulfonyl-anilino)prop-1-ynyl]-1-(2,2,2-trifluoroethyl)indol-4-amine 17

Compound 23: 4-((3-(4-(((3S,4R)-3-fluoro-1-methylpiperidin-4-yl)amino)-1-(2,2,2-trifluoroethyl)-1H-indol-2-yl)prop-2-yn-1-yl)amino)-3-methoxybenzenesulfonamide 18

Compound 24: 4-((3-(4-(((3S,4R)-3-fluoro-1-methylpiperidin-4-yl)amino)-1-(2,2,2-trifluoroethyl)-1H-indol-2-yl)prop-2-yn-1-yl)amino)-3-methoxybenzoic acid 18

Compound 25: 4-[3-[4-[[(3S,4R)-3-fluoro-1-methyl-4-piperidyl]amino]-1-(2,2,2-trifluoroethyl)indol-2-yl]prop-2-ynyl-amino]-3-methoxy-benzamide 19

Compound 27: 4-((3-(4-(((3S,4R)-3-fluoro-1-methylpiperidin-4-yl)amino)-1-(2,2,2-trifluoroethyl)-1H-indol-2-yl)prop-2-yn-1-yl)amino)-3-methoxy-N-(tetrahydro-2H-pyran-4-yl)benzamide 19

Compound 28: [4-[3-[4-[[(3S,4R)-3-fluoro-1-methyl-4-piperidyl]amino]-1-(2,2,2-trifluoroethyl)indol-2-yl]prop-2-ynyl-amino]-3-methoxy-phenyl]-(2-oxa-6-azaspiro[3.3]heptan-6-yl)methanone 20

Compound 29: N-(2,3-dihydroxypropyl)-4-[3-[4-[[(3S,4R)-3-fluoro-1-methyl-4-piperidyl]amino]-1-(2,2,2-trifluoroethyl)indol-2-yl]prop-2-ynylamino]-3-methoxy-benzamide 20

Compound 30: [4-[3-[4-[[(3S,4R)-3-fluoro-1-methyl-4-piperidyl]amino]-1-(2,2,2-trifluoroethyl)indol-2-yl]prop-2-ynyl-amino]-3-methoxy-phenyl]-(4-methylpiperazin-1-yl)methanone 21

Compound 31: 4-[3-[4-[[(3S,4R)-3-fluoro-1-methyl-4-piperidyl]amino]-1-(2,2,2-trifluoroethyl)indol-2-yl]prop-2-ynyl-amino]-3-methoxy-N-(1-methyl-4-piperidyl)benzamide 21

X-ray Crystallography Study 22

Time-Resolved Fluorescence Resonance Energy Transfer (TR-FRET) Assay 22

Cell Proliferation Assay 23

Pharmacokinetic Study in Mice 23

Study of Compound 26 in NUGC-3 Xenograft 24

General Information

All starting materials were commercially available and used without further purification unless noted otherwise. All compounds are >95% pure by liquid chromatography–mass spectrometry (LCMS). ^{1}H nuclear magnetic resonance (NMR) spectra were recorded on a Bruker spectrometer at indicated frequencies. Chemical shift (*δ* values) and coupling constants (*J* values) are given in ppm and Hertz, respectively.

Safety Statement

No unexpected or unusually high safety hazards were encountered while performing these experiments.

Preparation of Compound 13

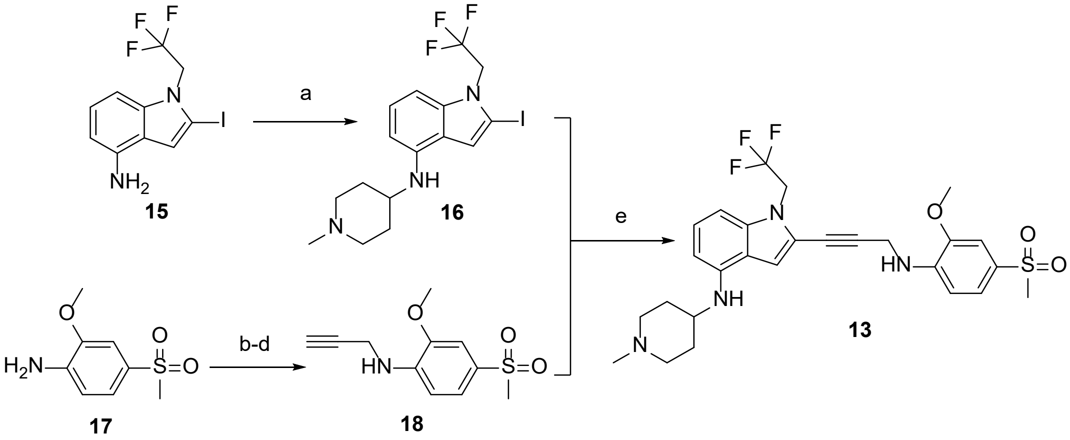

Reagents and conditions: (a) $SnCl_2$, $NaBH_3CN$, 1-methyl-4-piperidone; (b) Boc_2O, dioxane; (c) NaH, DMF, propargyl bromide; (d) EtOAc/HCl; (e) CuI, i-Pr_2NH, $Pd(PPh_3)_4$, DMSO

DMF, dimethylformamide; DMSO, dimethylsulfoxide.

2-Iodo-1-(2,2,2-trifluoroethyl)-1H-indol-4-amine **15** was prepared from 4-nitroindole as described in WO2017143291. ^{1}H NMR (400 MHz, dimethylsulfoxide [DMSO]-d_6) *δ* ppm 9.19–10.88 (m, 2 H), 7.63 (d, *J*=8.34 Hz, 1 H), 7.16–7.25 (m, 1 H), 7.04–7.14 (m, 2 H), 5.14–5.33 (m, 2 H). LCMS (ES^+, *m/z*): 340.9 [$(M+H)^+$].

$SnCl_2.2H_2O$ (398.11 mg, 1.76 mmol, 0.20 *eq.*) was added to a solution of 2-iodo-1-(2,2,2-trifluoroethyl)-1H-indol-4-amine 15 (3.00 g, 8.82 mmol, 1.00 *eq.*) and 1-methylpiperidin-4-one (1.20 g, 10.61 mmol, 1.20 *eq.*) in MeOH (10.00 mL). The mixture was stirred at 25 °C for 3 hours (h), and then $NaBH_3CN$ (2.77 g, 44.1 mmol, 5.00 *eq.*) was added, stirring at 25 °C for 69 h. Thin-layer chromatography (TLC) indicated that the starting material was consumed, and the reaction mixture was filtered. The filtrate was poured into H_2O (200 mL) and extracted with ethyl acetate ([EtOAc] 200 mL*2). The combined organic layers were washed with H_2O (200 mL), dried over Na_2SO_4, and concentrated under reduced pressure to give a residue. The crude material was purified by flash column chromatography (Silica gel, petroleum ether (PE) : EtOAc = 0:1) and then by preparative high performance chromatography ([prep-HPLC] column: Phenomenex Luna C18 100*40mm*5 um; mobile phase: [H_2O (0.2% Formic acid-acetonitrile [ACN])]; gradient: 10%–50% acetonitrile over 8.0 minutes) to yield 2-iodo-N-(1-methylpiperidin-4-yl)-1-(2,2,2-trifluoroethyl)-1H-indol-4-amine 16 (2.50 g, 5.72 mmol, 64.93% yield) as a light-brown solid.

^{1}H NMR (400 MHz, DMSO-d_6) δ ppm 8.24 (br s, 1 H, formic acid salt), 7.17 (s, 1 H), 6.85–6.95 (m, 1 H), 6.78 (br d, J = 7.99 Hz, 1 H), 6.16 (br d, J = 7.63 Hz, 1 H), 5.44 (br d, J = 2.62 Hz, 1 H), 4.99 (q, J = 8.54 Hz, 2 H), 3.33 (br s, 1 H), 2.85 (br d, J = 9.66 Hz, 2 H), 2.25 (br s, 3 H), 2.07–2.18 (m, 2 H), 1.94 (br d, J = 12.04 Hz, 2 H), 1.46–1.58 (m, 2 H). LCMS (ES$^+$, *m/z*): 438.0 [(M+H)$^+$].

Boc_2O (26.03 g, 119.26 mmol, 6.00 *eq.*) was added to a solution of 2-methoxy-4-(methylsulfonyl)aniline 17 (4.00 g, 19.88 mmol, 1.00 *eq.*) in dioxane (40.00 mL) at 25 °C (room temperature). The reaction mixture was stirred at 110 °C for 16 h. TLC and LCMS indicated that the reaction was completed, and it was concentrated in vacuo. The residue was purified by column chromatography (SiO_2, PE/EtOAc = 10/1 to 1:1) to yield tert-butyl (2-methoxy-4-(methylsulfonyl)phenyl)carbamate (6.00 g, 19.92 mmol, 72.6% purity, 72% yield) as a yellow gum.

^{1}H NMR (400 MHz, DMSO-d_6) δ ppm 8.33 (s, 1 H), 8.03 (d, J = 8.38 Hz, 1 H), 7.47 (dd, J = 8.38, 2.00 Hz, 1 H), 7.44 (d, J = 2.00 Hz, 1 H), 3.91 (s, 3 H), 3.18 (s, 3 H), 1.47 (s, 9 H). LCMS (ES$^+$, *m/z*): 324.1 [(M+Na)$^+$].

NaH (867.27 mg, 60% purity, 21.69 mmol, 3.00 *eq.*) was added in portions at 0 °C to a mixture of tert-butyl (2-methoxy-4-(methylsulfonyl)phenyl)carbamate (3.00 g, 7.23 mmol, 1.00 *eq.*) in dimethylformamide ([DMF] 30.00 mL) and stirred at 0 °C for 0.5 h. 3-Bromoprop-1-yne (3.23 g, 21.69 mmol, 3.00 *eq.*) was added to the reaction mixture, stirring at 0 °C for 2.5 h. TLC (Plate 1: PE : EtOAc = 1:1) and LCMS indicated that the starting material was consumed, and the product was detected. The reaction mixture was poured into a saturated solution of NH_4Cl (200 mL) at 0 °C and was extracted with EtOAc (200 mL*3). The combined organic phase was dried over Na_2SO_4, filtered, and concentrated

in vacuo. The residue was purified by column chromatography (SiO_2, PE : EtOAc = 5:1 to 1:2) to give tert-butyl (2-methoxy-4-(methylsulfonyl)phenyl)(prop-2-yn-1-yl)carbamate (3.00 g, 8.85 mmol, 74% purity, 90% yield) as a light-yellow gum.

^{1}H NMR (400 MHz, DMSO-d_6) δ ppm 7.53–7.56 (m, 1 H), 7.46–7.53 (m, 2 H), 4.10–4.51 (m, 2 H), 3.90 (s, 3 H), 3.27 (s, 3 H), 3.17 (t, J = 2.32 Hz, 1 H), 1.27–1.39 (m, 9 H). LCMS (ES^+, *m/z*): 283.9 [$(M+H\text{-}t\text{-}Bu)^+$].

A solution of 4M HCl/EtOAc (20.00 mL) was added to the solution of tert-butyl (2-methoxy-4-(methylsulfonyl)phenyl)(prop-2-yn-1-yl)carbamate (3.00 g, 6.54 mmol, 1.00 *eq.*) in EtOAc (1.00 mL). The reaction mixture was stirred at 25 °C for 2 h. TLC indicated that the starting material was consumed completely. The reaction mixture was concentrated in vacuo to yield 2-methoxy-4-(methylsulfonyl)-N-(prop-2-yn-1-yl)aniline 18 (1.80 g, 7.53 mmol, 85.3% yield, HCl salt) as a yellow solid.

^{1}H NMR (400 MHz, DMSO-d_6) δ ppm 7.38 (dd, J = 8.40, 1.60 Hz, 1 H), 7.22 (d, J = 1.60 Hz, 1 H), 6.75 (d, J = 8.80 Hz, 1 H), 3.99 (d, J = 2.4 Hz, 2 H), 3.87 (s, 3 H) 3.10 (s, 3 H), 3.08 (t, J = 2.31 Hz, 1 H). LCMS (ES^+, *m/z*): 240.1 [$(M+H)^+$].

i-Pr_2NH (2.08 g, 20.58 mmol, 2.91 mL, 10 *eq.*), CuI (392.02 mg, 2.06 mmol, 1 eq), 2-iodo-N-(1-methylpiperidin-4-yl)-1-(2,2,2-trifluoroethyl)-1H-indol-4-amine **16** (0.9 g, 2.06 mmol, 1 *eq.*) and $Pd(PPh_3)_4$ (475.71 mg, 411.67 μmol, 0.2 *eq.*) was added to a solution of 2-methoxy-4-(methylsulfonyl)-N-(prop-2-yn-1-yl)aniline **18** (622.16 mg, 2.47 mmol, 1.2 *eq.*) in DMSO (10 mL) at 45 °C under N_2. The reaction mixture was stirred at 45 °C for 1 h. TLC (DCM/MeOH=10:1, R_f = 0.3) indicated that the starting material was consumed completely. It was poured into ethylenediaminetetraacetic acid ([EDTA] 20 mL) and stirred for 1 h, then extracted with EtOAc (40 mL*3). The combined organic phase was washed with brine (40 mL), dried with anhydrous Na_2SO_4, filtered, and concentrated in vacuo. The crude product was purified by column chromatography (SiO_2, PE : EtOAc = 1:1 to dichloromethane (DCM) / MeOH = 10:1, R_f = 0.3), then by prep-HPLC (column: Phenomenex Luna(2) C18 250*50 10u; mobile phase: [water (0.1% trifluoroacetic acid)-ACN]; B%: 30%–50%, 20 min) to yield compound 13 (0.6 g, 1.09 mmol, 53.08% yield, 99.9% purity) as a light-yellow solid.

1 H NMR (400 MHz, DMSO-d_6) δ ppm 1.41–1.54 (m, 2 H), 1.91 (br d, J = 11.00 Hz, 2 H), 1.95–2.08 (m, 2 H) 2.17 (s, 3 H), 2.68–2.80 (m, 2 H), 3.10 (s, 3 H), 3.20–3.29 (m, 1 H), 3.89 (s, 3 H), 4.36 (d, J = 6.24 Hz, 2 H), 4.92 (q, J = 9.09 Hz, 2 H), 5.49 (d, J = 7.95 Hz, 1 H), 6.15 (d, J = 7.83 Hz, 1 H), 6.50 (t, J = 6.24 Hz, 1 H), 6.68 (d, J = 8.19 Hz, 1 H), 6.89 (d, J = 8.44 Hz, 1 H), 6.99 (t, J = 8.01 Hz, 1 H), 7.09 (s, 1 H), 7.25 (d, J = 1.83 Hz, 1 H), 7.39 (dd, J = 8.31, 1.83 Hz, 1 H). LCMS (ES^+, *m/z*): 549.3 [$(M+H)^+$].

Preparation of Compound 26[a]

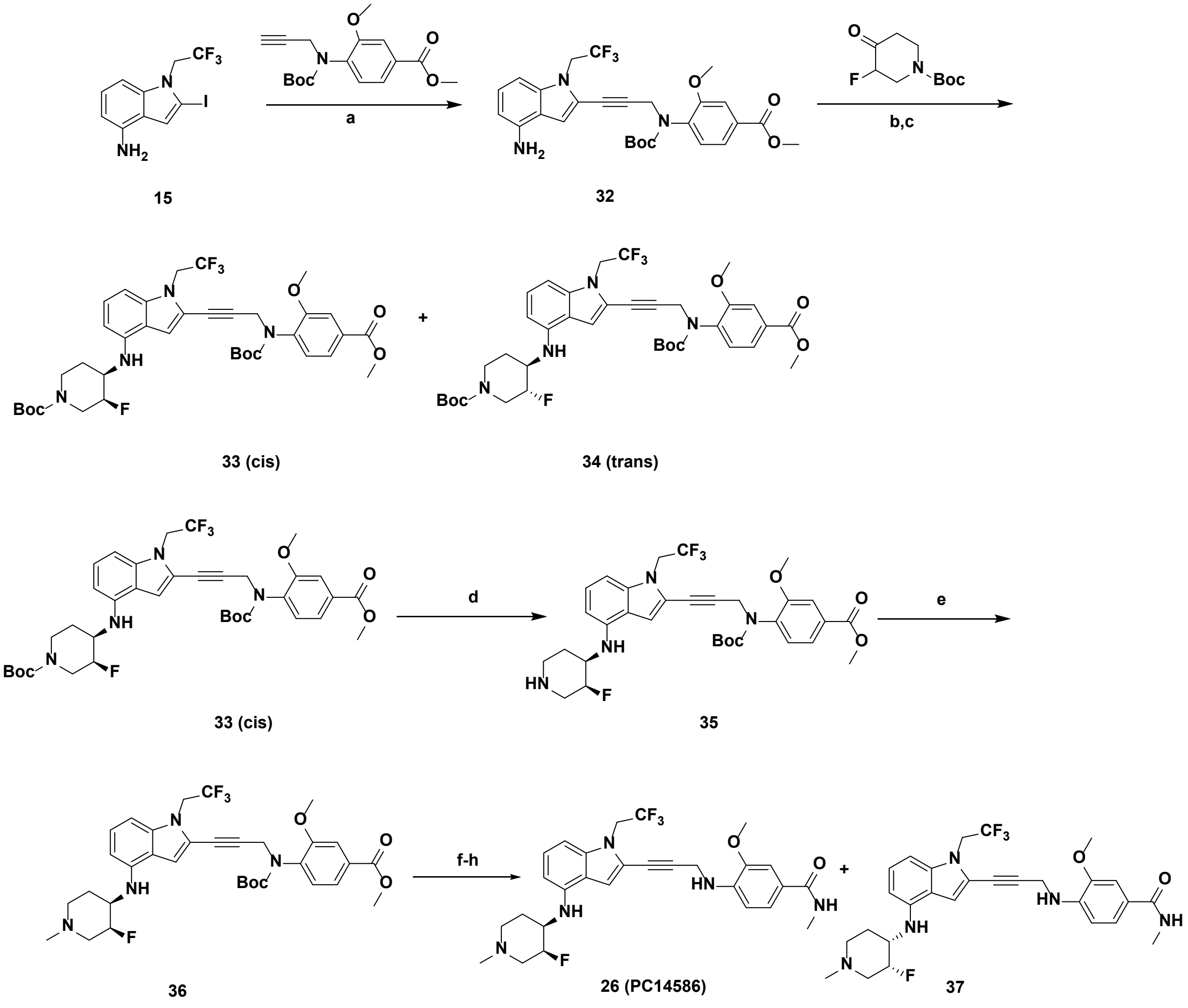

[a]Reagents and conditions: (a) Pd(PPh3)4, CuI, diisopropylamine, DMSO, 20 °C, 1 h; (b) TMSCl, DMF, 0 °C, 0.5 h; (c) BH3.THF, 0 °C, 0.5 h; (d) EtOAc/HCl, 20 °C, 1 h; (e) 10 *eq.* (CH2O)n, NaBH3CN, MeOH, 20 °C, 16 h; f) LiOH.H2O, MeOH, 40 °C, 12 h; g) MeNH3Cl, HOBT, EDCI, TEA, DCM, RT, 16 h; h) Chiral SFC separation

Methyl 4-((3-(4-amino-1-(2,2,2-trifluoroethyl)-lH-indol-2-yl)prop-2-yn-1-yl)(tert-butoxycarbonyl)amino)-3-methoxybenzoate (compound 32)

To a solution of methyl 4-((tert-butoxycarbonyl)(prop-2-yn-l-yl)amino)-3-methoxybenzoate (6.9 g, 21.39 mmol, 1.2 *eq.*) in DMSO (50 mL) were added CuI (1.02 g, 5.35 mmol, 0.3 *eq.*), diisopropylamine (18.04 g, 178.25 mmol, 25.19 mL, 10 *eq.*), $Pd(PPh_3)_4$ (1.03 g, 891.26 pmol, 0.05 *eq.*), and 2-iodo-l-(2,2,2-trifluoroethyl)-lH-indol-4-amine hydrochloride (6.06 g, 17.83 mmol, 1 *eq.*) under nitrogen. The reaction mixture was stirred for 1 h at 20 °C, after which time TLC analysis (PE:EtOAc = 2:1, Rf = 0.24) indicated that the reaction was complete. The reaction mixture was quenched with saturated aqueous solution of EDTA (500 mL) at 25 °C, stirred for 1 h, and then extracted with EtOAc (200 mL x 3). The combined organic layers were dried over anhydrous sodium sulfate, filtered, and concentrated under reduced pressure. The residue was purified by column chromatography (SiO_2, PE:EtOAc = 3:1 to 1:2, PE:EtOAc = 1:1, Rf = 0.24) to provide methyl 4-((3-(4-amino-1-(2,2,2-trifluoroethyl)-lH-indol-2-yl)prop-2-yn-1-yl)(tert-butoxycarbonyl)amino)-3-methoxybenzoate (10 g, 77% yield) as a brown oil.

(*Rac*) -tert-butyl (3S,4R)-4-((2-(3-((tert-butoxycarbonyl)(2-methoxy-4-(methoxycarbonyl)phenyl)amino)prop-2-yn1-yl)-1-(2,2,2-trifluoroethyl)-1H-indol-4-yl)amino)-3-fluoropiperidine-1-carboxylate and compound 34 (*rac*)-tert-butyl (3R,4R)-4-((2-(3-((tert-butoxycarbonyl)(2-methoxy- (methoxycarbonyl) phenyl) amino) prop-2-yn-1-yl)-1-(2,2,2-trifluoroethyl)-1H-indol-4-yl)amino)-3-fluoropiperidine-1-carboxylate (Compound 33)

To a solution of tert-butyl 3-fluoro-4-oxopiperidine-l-carboxylate (16 g, 73.65 mmol, 4 *eq.*) and methyl 4-((3-(4-amino-1-(2,2,2-trifluoroethyl)-1H-indol-2-yl)prop-2-yn-1 -yl)(tert-butoxycarbonyl)amino)-3-methoxybenzoate (9.79 g, 18.41 mmol, 1 *eq.*) in DMF (20 mL) was added TMSCl (6 g, 55.24 mmol, 7.01 mL, 3 *eq.*). The mixture was stirred at 0 °C for 0.5 h, where after BH_3-THF (1 M, 184 mL, 10 *eq.*) was added under N2. The mixture was stirred at 0 °C for an additional 0.5 h, after which time liquid chromatography-tandem mass spectrometry (LC-MS) analysis indicated that the starting primary amine was completely consumed. The reaction mixture was adjusted to pH~8 with saturated solution of Na_2CO_3, diluted with water (50 mL), and extracted with EtOAc (150 mL x 4). The combined organic layers were washed with brine (100 mL), dried over anhydrous sodium sulfate, filtered, and concentrated under reduced pressure. The residue was purified by prep-HPLC to provide (*rac*)-tert-butyl (3S,4S)-4-((2-(3-((tert-butoxycarbonyl)(2-methoxy-4-(methoxycarbonyl)phenyl)amino)prop-2-yn-1-yl)-l-(2,2,2-trifluoroethyl)-lH-indol-4-yl)amino)-3-fluoropiperidine-1-carboxylate (5.7 g, 7.78 mmol, 42.3% yield) as a yellow solid. The trans diastereomer was also isolated in 35% yield (4.7 g).

(*Rac*)-methyl 4-((3-(4-(((3S,4R)-3-fluoropiperidin-4-yl)amino)-1-(2,2,2-trifluoroethyl)-lH-indol-2-yl)prop-2-yn-1-yl)amino)-3-methoxybenzoate (Compound 35)

To a solution of (*rac*)-tert-butyl (3S.4R)-4-((2-(3-((tert-butoxycarbonyl)(2-methoxy-4-(methoxycarbonyl)phenyl)amino)prop-2 -yn-1-yl)-1-(2,2,2-trifluoroethyl)-1H-indol-4-yl)amino)-3-fluoropiperidine-1-carboxylate (5 g, 6.82 mmol, 1 *eq.*) was added 4N HCl/EtOAc (34.12 mmol, 20 mL, 5 *eq.*). The mixture was stirred at 20 °C for 1 h, after which time TLC analysis (DCM:MeOH = 10:1) indicated that the Boc protected starting material was completely consumed, and one new spot had appeared. The reaction mixture was adjusted to pH~8 with saturated solution of Na_2CO_3, diluted with water (50 mL), and extracted with EtOAc (50 mL x 4). The combined organic layers were washed with saturated solution of NaCl (10 mL), dried over anhydrous sodium sulfate, filtered, and concentrated under reduced pressure to provide crude (*rac*)-methyl 4-((3-(4-(((3S,4R)-3-fluoropiperidin-4-yl)amino)-1 -(2,2,2-trifluoroethyl)-1H-indol-2-yl)prop-2-yn-1-yl)amino)-3-methoxybenzoate (4.1 g, crude) as a yellow solid. It was used without further purification.

(*Rac*)-Methyl 4-((3-(4-(((3S,4R)-3-fluoro-1-methylpiperidin-4-yl)amino)-1-(2,2,2-trifluoroethyl)-1H-indol-2-yl)prop-2-yn-1-yl)amino)-3-methoxybenzoate (Compound 36)

A mixture of (*rac*)-methyl 4-((3-(4-(((3S,4R)-3-fluoropiperidin-4-yl)amino)-1-(2,2,2-trifluoroethyl)-1H-indol-2-yl)prop-2-yn-1-yl)amino)-3-methoxybenzoate (0.6 g, 1.13 mmol, 1 *eq.*), paraformaldehyde (338 mg, 11.27 mmol, 310.37 µL, 10 *eq.*), $NaBH_3CN$ (212.41 mg, 3.38 mmol, 3 *eq.*), and AcOH (68 mg, 1.13 mmol, 64 mL, 1 *eq.*) in MeOH (20 mL) was degassed and purged with nitrogen. The mixture was stirred at 20 °C for 2 h under nitrogen atmosphere, after which time TLC analysis (EtOAc:TEA = 10:1, Rf = 0.65) indicated that one new spot had appeared. The reaction mixture was quenched with saturated aqueous $NaHCO_3$ (30 mL), and then extracted with EtOAc (40 mL x 3). The combined organic layers were washed with brine (25 mL x 3), dried over anhydrous sodium sulfate, filtered, and concentrated under reduced pressure. The residue was purified by prep-TLC (SiO_2, EtOAc:TEA = 20:1) to provide (*rac*)-methyl 4-((3-(4-(((3S,4R)-3-fluoro-1-methylpiperidin-4-yl)amino)-1-(2,2,2-trifhioroethyl)-lH-indol-2-yl)prop-2-yn-l-yl)amino)-3-methoxybenzoate (0.4 g, 58% yield) as a yellow solid.

4-{[3-(4-{[(3S,4R)-3-fluoro-1-methylpiperidin-4-yl]amino}-1-(2,2,2-trifluoroethyl)-1H-indol-2-yl)prop-2-yn-1-yl]amino}-3-methoxy-N-methylbenzamide (Compound 26)

A mixture of (*rac*)-methyl 4-((3-(4-(((3S,4R)-3-fluoro-1-methylpiperidin-4-yl)amino)-1-(2,2,2-trifluoroethyl)-1H-indol-2-yl)prop-2-yn-1-yl)amino)-3-methoxybenzoate (0.4 g, 731.86 pmol, 1 *eq.*) in $LiOH.H_2O$ (10 mL, 10M)and MeOH (10 mL) was degassed and purged with nitrogen. The mixture was stirred at 40 °C for 12 h under nitrogen atmosphere, after which time TLC analysis (EtOAc:TEA = 10:1,

Rf = 0) indicated that one new spot had appeared. The reaction mixture was extracted with EtOAc (40 mL x 3). The combined organic layers were washed with brine (30 mL x 3), dried over anhydrous sodium sulfate, filtered, and concentrated under reduced pressure. The residue was purified by prep-TLC (SiO_2 , EtOAc:TEA = 10:1) to provide (*rac*)-4-((3-(4-(((3S,4R)-3-fluoro-1-methylpiperidin-4-yl)amino)-1-(2,2,2-trifluoroethyl)-lH-indol-2-yl)prop-2-yn-l-yl)amino)-3-methoxybenzoic acid (0.2 g, 46% yield) as a yellow solid.

A mixture of (*rac*)-4-((3-(4-(((3S,4R)-3-fluoro-1-methylpiperidin-4-yl)amino)-1-(2,2,2-trifluoroethyl)-1H-indol-2-yl)prop-2-yn-1-yl)amino)-3-methoxybenzoic acid (0.12 g, 225.3 mmol, 1 *eq.*), methylamine hydrochloride (30 mg, 450.68 mmol, 2 *eq.*), HOBt (46 mg, 338.01 mmol, 1.5 *eq.*), EDCI (65 mg, 338.01 mmol, 1.5 *eq.*), and TEA (91 mg, 901.36 mmol, 125.46 µL, 4 *eq.*) in DCM (5 mL) was degassed and purged with nitrogen. The mixture was stirred at room temperature for 16 h under nitrogen atmosphere, after which time TLC analysis (EtOAc:TEA = 10:1, Rf = 0.2) indicated that one new major spot had formed. The reaction mixture was diluted with EtOAc (15 mL) and extracted with EtOAc (20 mL x 3). The combined organic layers were washed with brine (20 mL x 3), dried over anhydrous sodium sulfate, filtered, and concentrated under reduced pressure. The residue was purified by prep-TLC (SiO_2, EtOAc:TEA = 10:1), and further purified by prep-HPLC to provide (*rac*)-4-{ [3-(4-([(3S,4R)-3-fluoro-l-methylpiperidin -4-yljamino} -1-(2,2,2-trifluoroethyl)-lH-indol-2-yl)prop-2-yn-l-yl]amino}-3-methoxy-N-methylbenzamideas a yellow solid (22.1 mg, 18.0% yield). LC- MS (ES^+,*m/z*): 546.3

Chiral SFC Separation: (condition: column: DAICEL CHIRALCEL OJ (250mm*30mm,10um); mobile phase: [0.1% NH_3H_2O EtOH]; B%: 35%-35%, 3.2min). Compound 26 (peak 1) was obtained as a yellow solid. 1H NMR: (400 MHz, DMSO-d_6) δ ppm 1.65 - 1.73 (1 H, m) 1.86 - 1.98 (1 H, m) 2.04 - 2.12 (1 H, m) 2.15 (4 H, br s) 2.73 - 2.82 (4 H, m) 2.97 - 3.07 (1 H, m) 3.49 - 3.63 (1 H, m) 3.80 - 3.87 (3 H, m) 4.26 - 4.35 (2 H, m) 4.72 - 4.96 (3 H, m) 5.45 - 5.53 (1 H, m) 5.95 - 6.03 (1 H, m) 6.20 - 6.27 (1 H, m) 6.70 - 6.78 (2 H, m) 6.97 - 7.04 (1 H, m) 7.13 - 7.19 (1 H, m) 7.32 - 7.36 (1 H, m) 7.39 - 7.45 (1 H, m) 8.07 - 8.14 (1 H, m).

List of compounds and structures

Compound 1: N-[3-[1-ethyl-5-(methylaminomethyl)indol-2-yl]prop-2-ynyl]aniline

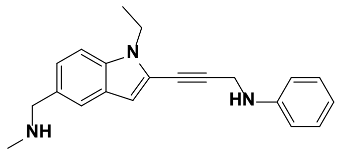

^{1}H NMR (400 MHz, MeOD-d_4) δ ppm 7.69 (s, 1 H), 7.56–7.58 (d, J = 8.0 Hz, 2 H), 7.48–7.52 (m, 4 H), 7.35–7.37 (d, J = 8.0 Hz, 1 H), 6.84 (s, 1 H), 4.68 (s, 2 H), 4.20–4.30 (m, 4 H), 2.76 (s, 3 H), 1.25 (t, J = 8.0 Hz, 3 H). LCMS (ES$^+$, m/z): 287.2 [(M+H)$^+$].

Compound 2: 2-(3-anilinoprop-1-ynyl)-1-ethyl-N-(1-methyl-4-piperidyl)indole-5-carboxamide

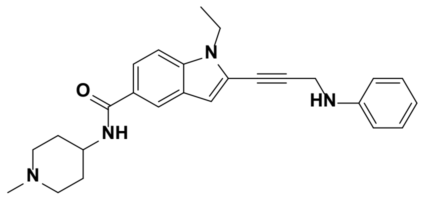

^{1}H NMR (400 MHz, MeOD-d_4) δ ppm 8.02 (s, 1 H), 7.66 (d, J = 1.6 Hz, 1 H), 7.20 (d, J = 7.2 Hz, 1 H), 7.16–7.18 (m, 2 H), 6.81–6.83 (m, 2 H), 6.69–6.75 (m, 2 H), 4.24 (s, 2 H), 4.12–4.16 (m, 2 H), 3.89–3.90 (m, 1 H), 2.90–2.93 (m, 2 H), 2.30 (s, 3 H), 2.17–2.20 (m, 2 H), 1.94–1.97 (m, 2 H), 1.66–1.70 (m, 2 H), 1.16 (t, J = 6.8 Hz, 3 H). LCMS (ES$^+$, m/z): 415.3 [(M+H)$^+$]

Compound 3: N-((1-ethyl-2-(3-(phenylamino)prop-1-yn-1-yl)-1H-indol-5-yl)methyl)tetrahydro-2H-pyran-4-amine

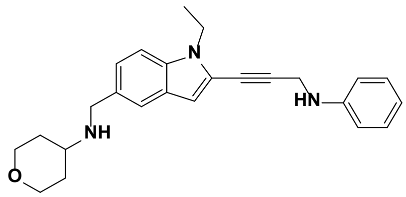

^{1}H NMR (400 MHz, MeOD-d_4) δ ppm 7.71 (s, 1 H), 7.47–7.57 (m, 3 H), 7.35–7.47 (m, 4 H), 6.81 (s, 1 H), 4.64 (s, 2 H), 4.31 (s, 2 H), 4.21 (q, J = 7.1 Hz, 2 H), 4.04 (br dd, J = 4.1, 11.8 Hz, 2 H), 3.44 (br t, J =11.8 Hz, 3 H), 2.11 (br d, J = 10.1 Hz, 2 H), 1.71 (br dd, J = 4.5, 12.0 Hz, 2 H), 1.23 (t, J = 7.1 Hz, 3 H). LCMS (ES$^+$, m/z): 388.3 [(M+H)$^+$].

Compound 4: N-(3-(1-ethyl-5-(((tetrahydro-2H-pyran-4-yl)amino)methyl)-1H-indol-2-yl)prop-2-yn-1-yl)-6-methylpyridin-3-amine

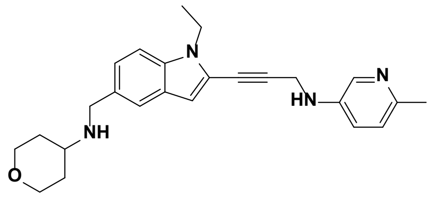

^{1}H NMR (400 MHz, MeOD-d_4) δ ppm 8.02 (d, J = 2.9 Hz, 1 H), 7.85 (dd, J = 2.9, 9.0 Hz, 1 H), 7.65–7.69 (m, 2 H), 7.48 (d, J = 8.6 Hz, 1 H), 7.35 (dd, J = 1.8, 8.6 Hz, 1 H), 6.74 (s, 1 H), 4.43 (s, 2 H), 4.27–4.33 (m, 4 H), 4.04 (br dd, J = 4.2, 11.5 Hz, 2 H), 3.44 (dt, J = 2.0, 12.0 Hz, 3 H), 2.62 (s, 3 H), 2.05–2.15 (m, 2 H), 1.71 (br dd, J = 4.6, 12.6 Hz, 2 H), 1.27 (t, J = 7.2 Hz, 3 H). LCMS (ES$^+$, *m/z*): 403.3 [(M+H)$^+$].

Compound 5: 6-(tert-butyl)-3-(3-{1-ethyl-5-[(methylamino)methyl]-2-indolyl}-2-propynylamino)pyridine

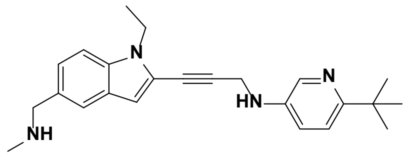

^{1}H NMR (400 MHz, MeOD-d_4) δ ppm 8.05 (d, J = 2.43 Hz, 1 H), 7.61 (d, J = 1.10 Hz, 1 H), 7.42 (d, J = 8.60 Hz, 1 H), 7.31 (d, J = 8.60 Hz, 1 H), 7.26 (dd, J = 8.49, 1.65 Hz, 1 H), 7.20 (dd, J = 8.60, 2.87 Hz, 1 H), 6.67 (s, 1 H), 4.29 (s, 2 H), 4.13–4.21 (m, 4 H), 2.67 (s, 3 H), 1.33 (s, 9 H), 1.11–1.17 (m, 3 H). LCMS (ES$^+$, *m/z*): 375.3 [(M+H)$^+$].

Compound 6: 2-(5-((3-(1-ethyl-5-(((tetrahydro-2H-pyran-4-yl)amino)methyl)-1H-indol-2-yl)prop-2-yn-1-yl)amino)pyridin-2-yl)-2-methylpropanenitrile

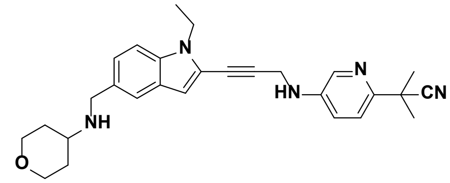

^{1}H NMR (400 MHz, MeOD-d_4) δ ppm 8.53 (s, 1 H), 8.12 (d, J = 2.8 Hz, 1 H), 7.63 (s, 1 H), 7.43 (d, J = 8.5 Hz, 2 H), 7.29 (dd, J = 1.5, 8.5 Hz, 1 H), 7.24 (dd, J = 2.9, 8.5 Hz, 1 H), 6.68 (s, 1 H), 4.32 (s, 2 H), 4.25 (s, 2 H), 4.19 (q, J = 7.2 Hz, 2 H), 4.02 (br dd, J = 4.4, 11.5 Hz, 2 H), 3.37–3.47 (m, 2 H), 2.07

(br dd, J = 2.2, 12.4 Hz, 2 H), 1.61–1.75 (m, 8 H), 1.16 (t, J = 7.2 Hz, 3 H). LCMS (ES^+, *m/z*): 456.3 [$(M+H)^+$]

Compound 7: 2-methyl-2-(5-((3-(5-(((tetrahydro-2H-pyran-4-yl)amino)methyl)-1-(2,2,2-trifluoroethyl)-1H-indol-2-yl)prop-2-yn-1-yl)amino)pyridin-2-yl)propanenitrile

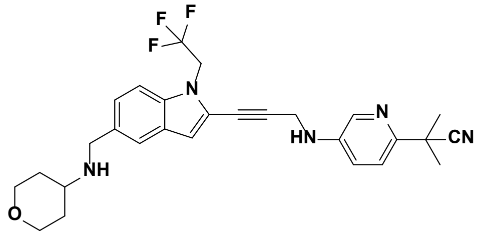

^{1}H NMR (400 MHz, MeOD-d_4) δ ppm 8.52 (s, 1 H), 8.08–8.13 (m, 1 H), 7.68 (d, J = 1.1 Hz, 1 H), 7.51 (d, J = 8.6 Hz, 1 H), 7.36–7.42 (d, J = 8.6 Hz, 2 H), 7.23 (dd, J = 2.9, 8.6 Hz, 1 H), 6.82 (s, 1 H), 4.88–4.94 (m, 2 H), 4.33 (s, 2 H), 4.26 (s, 2 H), 4.03 (dd, J = 4.4, 11.5 Hz, 2 H), 3.43 (dt, J = 1.8, 12.0 Hz, 2 H), 3.34–3.38 (m, 1 H), 2.08 (br dd, J = 2.2, 12.3 Hz, 2 H), 1.61–1.75 (m, 8 H). LCMS (ES^+, *m/z*): 510.2 [$(M+H)^+$].

Compound 8: 2-methyl-2-(5-((3-(4-(((tetrahydro-2H-pyran-4-yl)amino)methyl)-1-(2,2,2-trifluoroethyl)-1H-indol-2-yl)prop-2-yn-1-yl)amino)pyridin-2-yl)propanenitrile

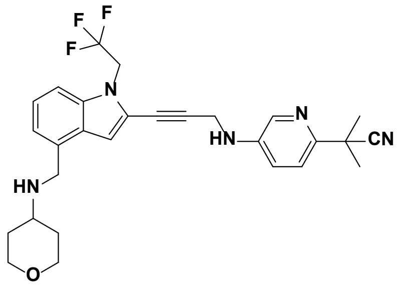

^{1}H NMR (400 MHz, MeOD-d4) δ ppm 8.51–8.58 (m, 1 H, Formic acid salt), 8.10 (d, J = 2.87 Hz, 1 H), 7.40–7.47 (m, 2 H), 7.31 (t, J = 7.83 Hz, 1 H), 7.18–7.26 (m, 2 H), 6.98 (s, 1 H), 4.89–4.95 (m, 2 H), 4.34 (s, 2 H), 4.26 (s, 2 H), 4.00 (br dd, J = 11.80, 4.08 Hz, 2 H), 3.41 (t, J = 11.36 Hz, 2 H), 3.09–3.21 (m, 1 H), 1.96–2.07 (m, 2 H), 1.71 (s, 6 H), 1.59 (qd, J = 12.27, 4.19 Hz, 2 H). LCMS (ES^+, *m/z*): 510.3 [(M+H)+].

Compound 9: 2-methyl-2-(5-((3-(4-((tetrahydro-2H-pyran-4-yl)amino)-1-(2,2,2-trifluoroethyl)-1H-indol-2-yl)prop-2-yn-1-yl)amino)pyridin-2-yl)propanenitrile

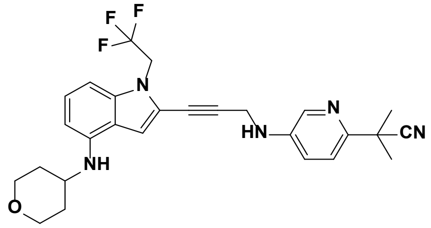

^{1}H NMR (400 MHz, MeOD-d_4) δ ppm 8.09 (d, J = 2.9 Hz, 1 H), 7.42 (d, J = 8.6 Hz, 1 H), 7.22 (dd, J = 2.9, 8.6 Hz, 1 H), 7.07 (t, J = 8.0 Hz, 1 H), 6.93 (s, 1 H), 6.68 (d, J = 8.2 Hz, 1 H), 6.34 (d, J = 7.7 Hz, 1 H), 4.74 (q, J = 8.8 Hz, 2 H), 4.30 (s, 2 H), 3.96–4.02 (m, 2 H), 3.61–3.68 (m, 1 H), 3.55 (dt, J = 1.9, 11.6 Hz, 2 H), 2.01–2.06 (m, 2 H), 1.71 (s, 6 H), 1.51–1.64 (m, 2 H). LCMS (ES$^+$, *m/z*): 496.3 [(M+H)$^+$].

Compound 10: 2-methyl-2-(5-((3-(4-((1-methylpiperidin-4-yl)amino)-1-(2,2,2-trifluoroethyl)-1H-indol-2-yl)prop-2-yn-1-yl)amino)pyridin-2-yl)propanenitrile

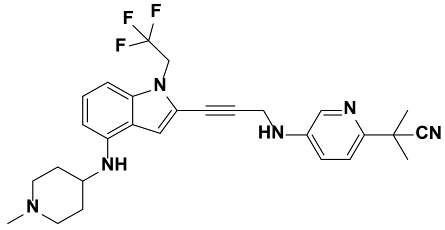

^{1}H NMR (400 MHz, MeOD-d_4) δ ppm 8.45–8.63 (m, 1 H), 8.09 (d, J = 2.4 Hz, 1 H), 7.42 (d, J = 8.6 Hz, 1 H), 7.22 (dd, J = 3.1, 8.6 Hz, 1 H), 7.08 (t, J = 8.0 Hz, 1 H), 6.93 (s, 1 H), 6.72 (d, J = 8.2 Hz, 1 H), 6.32 (d, J = 7.7 Hz, 1 H), 4.73–4.80 (m, 2 H), 4.31 (s, 2 H), 3.63–3.71 (m, 1 H), 3.34–3.40 (m, 2 H), 2.89–2.99 (m, 2 H), 2.73 (s, 3 H), 2.24 (br d, J = 12.1 Hz, 2 H), 1.75–1.83 (m, 2 H), 1.71 (s, 6H). LCMS (ES$^+$, *m/z*): 509.3 [(M+H)$^+$].

Compound 11: N-(1-methylpiperidin-4-yl)-2-(3-((4-(methylsulfonyl)phenyl)amino)prop-1-yn-1-yl)-1-(2,2,2-trifluoroethyl)-1H-indol-4-amine

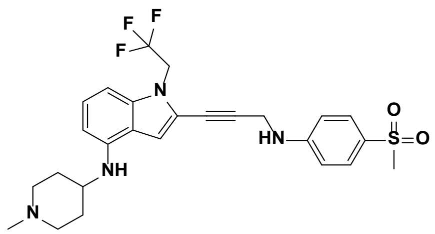

^{1}H NMR (400 MHz, MeOD-d_4) δ ppm 8.52 (br s, 1 H), 7.68–7.73 (m, 2 H), 7.09 (t, J = 8.0 Hz, 1 H), 6.94 (s, 1 H), 6.85–6.90 (m, 2 H), 6.72 (d, J = 8.2 Hz, 1 H), 6.33 (d, J = 7.7 Hz, 1 H), 4.73–4.79 (m, 2 H), 4.32 (s, 2 H), 3.68–3.77 (m, 1 H), 3.44 (br d, J = 12.3 Hz, 2 H), 3.02–3.12 (m, 5 H), 2.81 (s, 3 H), 2.27 (br d, J = 11.9 Hz, 2 H), 1.82 (br d, J = 9.9 Hz, 2 H). LCMS (ES$^+$, m/z): 519.3 [(M+H)$^+$].

Compound 12: 2-[3-(2-methyl-4-methylsulfonyl-anilino)prop-1-ynyl]-N-(1-methyl-4-piperidyl)-1-(2,2,2-trifluoroethyl)indol-4-amine

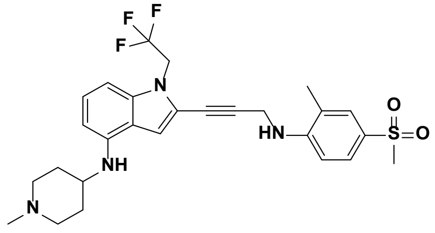

^{1}H NMR (400 MHz, DMSO-d_6) δ ppm 8.21 (s, 1 H), 7.58 (dd, J = 2.1, 8.6 Hz, 1 H), 7.52 (d, J = 1.7 Hz, 1 H), 7.09 (s, 1 H), 7.00 (t, J = 8.0 Hz, 1 H), 6.90 (d, J = 8.7 Hz, 1 H), 6.68 (d, J = 8.3 Hz, 1 H), 6.42–6.49 (m, 1 H), 6.16 (d, J = 7.8 Hz, 1 H), 5.49 (br d, J = 7.3 Hz, 1 H), 4.92 (br d, J = 9.2 Hz, 2 H), 4.37 (d, J = 5.9 Hz, 2 H), 3.30–3.33 (m, 1 H), 3.05 (s, 3 H), 2.81 (br d, J = 11.7 Hz, 2 H), 2.21 (s, 3 H), 2.17 (s, 3 H), 2.05–2.12 (m, 2 H), 1.92 (br d, J = 11.5 Hz, 2 H), 1.45–1.54 (m, 2 H). LCMS (ES$^+$, m/z): 533.1 [(M+H)$^+$].

Compound 14: N-(1-methyl-4-piperidyl)-2-[3-[4-methylsulfonyl-2-(trifluoromethoxy)anilino]prop-1-ynyl]-1-(2,2,2-trifluoroethyl)indol-4-amine

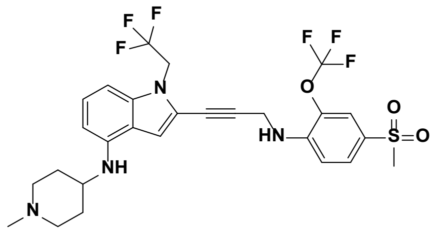

^{1}H NMR (400 MHz, DMSO-d_6) δ ppm 8.14–8.30 (m, 1 H), 7.74 (br d, J = 8.6 Hz, 1 H), 7.66 (s, 1 H), 7.30 (br t, J = 4.9 Hz, 1 H), 7.17 (d, J = 8.8 Hz, 1 H), 7.11 (s, 1 H), 6.97–7.04 (m, 1 H), 6.68 (d, J = 8.4 Hz, 1 H), 6.16 (d, J = 7.5 Hz, 1 H), 5.49–5.56 (m, 1 H), 4.86–4.96 (m, 2 H), 4.41 (br d, J = 5.7 Hz, 2 H), 3.33 (dt, J = 3.3, 5.6 Hz, 1 H), 3.15 (s, 3 H), 2.82–2.90 (m, 2 H), 2.25 (br s, 3 H), 2.11–2.20 (m, 2 H), 1.89–1.98 (m, 2 H), 1.46–1.58 (m, 2 H). LCMS (ES$^+$, m/z): 603.1 [(M+H)$^+$].

Compound 19: ***Rac*-N-[(3R,4R)-3-fluoro-1-methyl-4-piperidyl]-2-[3-(2-methoxy-4-methylsulfonyl-anilino)prop-1-ynyl]-1-(2,2,2-trifluoroethyl)indol-4-amine**

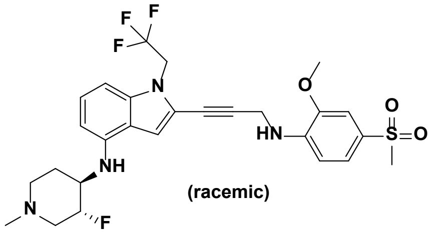

^{1}H NMR (400 MHz, DMSO-d_6) δ ppm 7.39 (dd, J = 1.8, 8.3 Hz, 1 H), 7.26 (d, J = 2.0 Hz, 1 H), 7.07 (s, 1 H), 7.00 (t, J = 7.9 Hz, 1 H), 6.90 (d, J = 8.4 Hz, 1 H), 6.70 (d, J = 8.2 Hz, 1 H), 6.50 (t, J = 6.2 Hz, 1 H), 6.24 (d, J = 7.8 Hz, 1 H), 5.70 (d, J = 8.3 Hz, 1 H), 4.93 (q, J = 9.1 Hz, 2 H), 4.45–4.63 (m, 1 H), 4.36 (d, J = 6.2 Hz, 2 H), 3.90 (s, 3 H), 3.46–3.56 (m, 1 H), 3.04–3.14 (m, 4 H), 2.70 (br s, 1 H), 2.23 (s, 3 H), 1.97–2.14 (m, 3 H), 1.39–1.51 (m, 1 H). LCMS (ES$^+$, *m/z*): 567.2 [(M+H)$^+$].

Compound 20: ***Rac*-N-[(3S,4R)-3-fluoro-1-methyl-4-piperidyl]-2-[3-(2-methoxy-4-methylsulfonyl-anilino)prop-1-ynyl]-1-(2,2,2-trifluoroethyl)indol-4-amine**

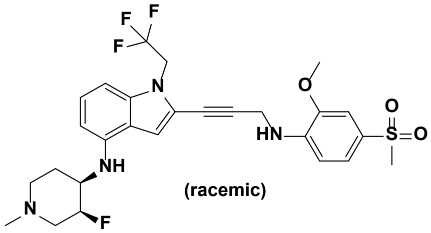

^{1}H NMR (400 MHz, DMSO-d_6) δ ppm 7.39 (dd, J = 1.9, 8.4 Hz, 1 H), 7.25 (d, J = 2.0 Hz, 1 H), 7.19 (s, 1 H), 7.01 (t, J = 8.0 Hz, 1 H), 6.89 (d, J = 8.4 Hz, 1 H), 6.74 (d, J = 8.2 Hz, 1 H), 6.49 (t, J = 6.3 Hz, 1 H), 6.24 (d, J = 7.8 Hz, 1 H), 5.49 (d, J = 8.6 Hz, 1 H), 4.71–5.00 (m, 3 H), 4.36 (d, J = 6.2 Hz, 2 H), 3.89 (s, 3 H), 3.48–3.67 (m, 1 H), 3.09 (s, 3 H), 2.99–3.07 (m, 1 H), 2.80 (br d, J = 11.2 Hz, 1 H), 2.15–2.30 (m, 4 H), 2.04–2.13 (m, 1 H), 1.85–2.01 (m, 1 H), 1.62–1.75 (m, 1 H). LCMS (ES$^+$, *m/z*): 567.2 [(M+H)$^+$].

Compound 21: N-[(3R,4S)-3-fluoro-1-methyl-4-piperidyl]-2-[3-(2-methoxy-4-methylsulfonyl-anilino)prop-1-ynyl]-1-(2,2,2-trifluoroethyl)indol-4-amine

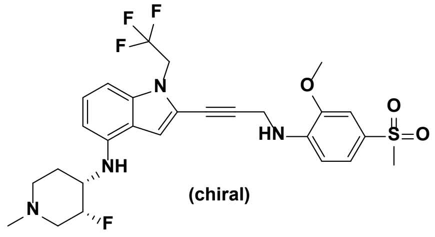

^{1}H NMR (400 MHz, DMSO-d_6) δ ppm 7.39 (dd, J = 2.0, 8.3 Hz, 1 H), 7.25 (d, J = 2.0 Hz, 1 H), 7.19 (s, 1 H), 6.97–7.06 (m, 1 H), 6.89 (d, J = 8.4 Hz, 1 H), 6.74 (d, J = 8.4 Hz, 1 H), 6.49 (t, J = 6.2 Hz, 1 H), 6.24 (d, J = 7.9 Hz, 1 H), 5.49 (br d, J = 8.6 Hz, 1 H), 4.73 - 5.01 (m, 3 H), 4.36 (d, J = 6.2 Hz, 2 H), 3.87–3.95 (m, 3 H), 3.50–3.67 (m, 1 H), 2.98–3.16 (m, 4 H), 2.76–2.86 (m, 1 H), 2.06–2.31 (m, 5 H), 1.85–1.99 (m, 1 H), 1.70 (br dd, J = 2.8, 12.7 Hz, 1 H). LCMS (ES$^+$, m/z): 567.2 [(M+H)$^+$].

Compound 22: N-[(3S,4R)-3-fluoro-1-methyl-4-piperidyl]-2-[3-(2-methoxy-4-methylsulfonyl-anilino)prop-1-ynyl]-1-(2,2,2-trifluoroethyl)indol-4-amine

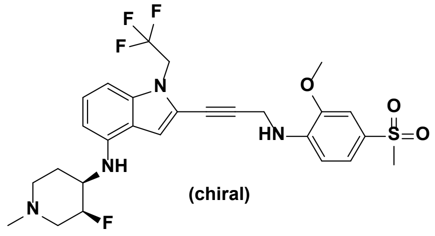

^{1}H NMR (400 MHz, DMSO-d_6) δ ppm 7.39 (dd, J = 2.0, 8.3 Hz, 1 H), 7.25 (d, J = 2.0 Hz, 1 H), 7.19 (s, 1 H), 6.97–7.06 (m, 1 H), 6.89 (d, J = 8.4 Hz, 1 H), 6.74 (d, J = 8.4 Hz, 1 H), 6.49 (t, J = 6.2 Hz, 1 H), 6.24 (d, J = 7.9 Hz, 1 H), 5.49 (br d, J = 8.6 Hz, 1 H), 4.73–5.01 (m, 3 H), 4.36 (d, J = 6.2 Hz, 2 H), 3.87–3.95 (m, 3 H), 3.50–3.67 (m, 1 H), 2.98–3.16 (m, 4 H), 2.76–2.86 (m, 1 H), 2.06–2.31 (m, 5 H), 1.85–1.99 (m, 1 H), 1.70 (br dd, J = 2.8, 12.7 Hz, 1 H). LCMS (ES$^+$, m/z): 567.2 [(M+H)$^+$].

Compound 23: 4-((3-(4-(((3S,4R)-3-fluoro-1-methylpiperidin-4-yl)amino)-1-(2,2,2-trifluoroethyl)-1H-indol-2-yl)prop-2-yn-1-yl)amino)-3-methoxybenzenesulfonamide

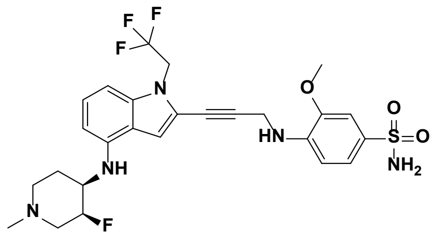

^{1}H NMR (400 MHz, DMSO-d_6) δ 7.31 (dd, J = 1.8, 8.3 Hz, 1 H), 7.25 (d, J = 1.8 Hz, 1 H), 7.18 (s, 1 H), 6.97–7.04 (m, 3 H), 6.81 (d, J = 8.4 Hz, 1 H), 6.74 (d, J = 8.3 Hz, 1 H), 6.18–6.28 (m, 2 H), 5.51 (d, J = 8.8 Hz, 1 H), 4.95 (q, J = 9.2 Hz, 2 H), 4.70–4.87 (m, 1 H), 4.33 (d, J = 6.2 Hz, 2 H), 3.85 (s, 3 H), 3.47–3.64 (m, 1 H), 2.97–3.09 (m, 1 H), 2.80 (br d, J = 11.4 Hz, 1 H), 2.18 (s, 4 H), 2.08 (br t, J = 11.4 Hz, 1 H), 1.85–1.97 (m, 1 H), 1.63–1.74 (m, 1 H). LCMS (ES$^+$, *m/z*): 568.3 [(M+H)$^+$].

Compound 24: 4-((3-(4-(((3S,4R)-3-fluoro-1-methylpiperidin-4-yl)amino)-1-(2,2,2-trifluoroethyl)-1H-indol-2-yl)prop-2-yn-1-yl)amino)-3-methoxybenzoic acid

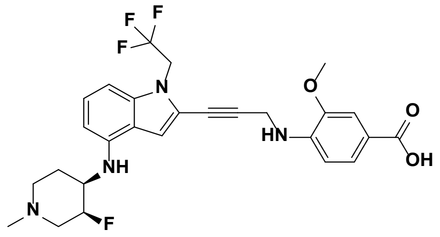

^{1}H NMR (400 MHz, DMSO-d_6) δ 12.22 (br s, 1 H), 8.14 (s, 1 H), 7.52 (dd, J = 1.7, 8.3 Hz, 1 H), 7.33 (d, J = 1.7 Hz, 1 H), 7.18 (s, 1 H), 7.00 (t, J = 8.0 Hz, 1 H), 6.79 (d, J = 8.3 Hz, 1 H), 6.73 (d, J = 8.3 Hz, 1 H), 6.29 (t, J = 6.3 Hz, 1 H), 6.24 (d, J = 7.8 Hz, 1 H), 5.50 (br d, J = 8.6 Hz, 1 H), 4.70–5.05 (m, 3 H), 4.34 (d, J = 6.2 Hz, 2 H), 3.84 (s, 3 H), 3.49–3.65 (m, 1 H), 3.05 (br t, J = 10.6 Hz, 1 H), 2.82 (br d, J = 10.1 Hz, 1 H), 2.03–2.28 (m, 5 H), 1.85–2.01 (m, 1 H), 1.64–1.75 (m, 1 H); LCMS (ES$^+$, *m/z*): 533.2 [(M+H)$^+$].

Compound 25: 4-[3-[4-[[(3S,4R)-3-fluoro-1-methyl-4-piperidyl]amino]-1-(2,2,2-trifluoroethyl)indol-2-yl]prop-2-ynyl-amino]-3-methoxy-benzamide

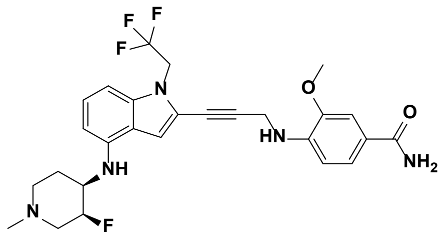

^{1}H NMR (400 MHz, DMSO-d_6) δ 7.67 (br s, 1 H), 7.46 (dd, J = 1.7, 8.3 Hz, 1 H), 7.38 (d, J = 1.8 Hz, 1 H), 7.17 (s, 1 H), 6.93–7.04 (m, 2 H), 6.70–6.77 (m, 2 H), 6.23 (d, J = 7.8 Hz, 1 H), 6.00 (t, J = 6.3 Hz, 1 H), 5.49 (d, J = 8.6 Hz, 1 H), 4.72–4.99 (m, 3 H), 4.32 (d, J = 6.4 Hz, 2 H), 3.84 (s, 3 H), 3.49–3.64 (m, 1 H), 2.97–3.09 (m, 1 H), 2.80 (br d, J = 10.5 Hz, 1 H), 2.14–2.29 (m, 4 H), 2.03–2.12 (m, 1 H), 1.92 (dq, J = 3.8, 12.1 Hz, 1 H), 1.63–1.72 (m, 1 H). LCMS (ES$^+$, *m/z*): 532.2 [$(M+H)^+$].

Compound 27: 4-((3-(4-(((3S,4R)-3-fluoro-1-methylpiperidin-4-yl)amino)-1-(2,2,2-trifluoroethyl)-1H-indol-2-yl)prop-2-yn-1-yl)amino)-3-methoxy-N-(tetrahydro-2H-pyran-4-yl)benzamide

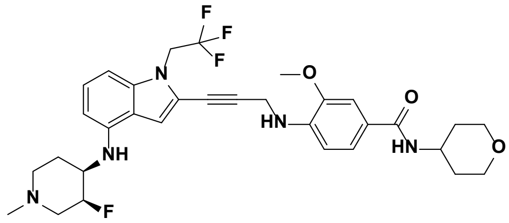

^{1}H NMR (400 MHz, DMSO-d_6) δ 8.15 (s, 0.4 H), 7.94 (d, J = 7.8 Hz, 1 H), 7.46 (dd, J = 1.6, 8.3 Hz, 1 H), 7.35 (d, J = 1.7 Hz, 1 H), 7.16 (s, 1 H), 7.00 (t, J = 8.0 Hz, 1 H), 6.71–6.78 (m, 2 H), 6.23 (d, J = 7.8 Hz, 1 H), 6.00 (t, J = 6.3 Hz, 1 H), 5.50 (br d, J = 8.6 Hz, 1 H), 4.72–4.98 (m, 3 H), 4.32 (d, J = 6.4 Hz, 2 H), 3.97 (dt, J = 3.5, 7.5 Hz, 1 H), 3.81–3.91 (m, 5 H), 3.49–3.64 (m, 1 H), 3.37 (br dd, J = 10.0, 11.8 Hz, 2 H), 2.99–3.08 (m, 1 H), 2.81 (br d, J = 11.0 Hz, 1 H), 2.17–2.31 (m, 4 H), 2.10 (br t, J = 11.2 Hz, 1 H), 1.92 (br dd, J = 3.4, 12.4 Hz, 1 H), 1.66–1.77 (m, 3 H), 1.57 (dq, J = 4.5, 12.0 Hz, 2 H). LCMS (ES$^+$, *m/z*): 616.3 [$(M+H)^+$].

Compound 28: [4-[3-[4-[[(3S,4R)-3-fluoro-1-methyl-4-piperidyl]amino]-1-(2,2,2-trifluoroethyl)indol-2-yl]prop-2-ynyl-amino]-3-methoxy-phenyl]-(2-oxa-6-azaspiro[3.3]heptan-6-yl)methanone

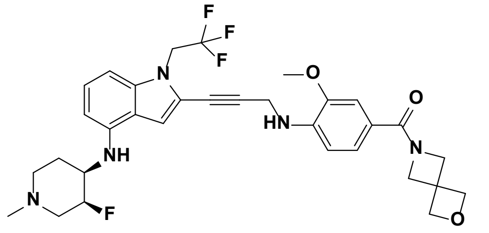

^{1}H NMR (400 MHz, DMSO-d_6) δ 8.29 (br d, J = 0.7 Hz, 1 H), 7.13–7.22 (m, 2 H), 7.10 (s, 1 H), 7.01 (br t, J = 7.8 Hz, 1 H), 6.68–6.80 (m, 2 H), 6.24 (br d, J = 7.7 Hz, 1 H), 6.09 (br t, J = 5.8 Hz, 1 H), 5.48 (br d, J = 8.8 Hz, 1 H), 4.73–4.99 (m, 3 H), 4.68 (s, 4 H), 4.36–4.55 (m, 2 H), 4.32 (br d, J = 5.9 Hz, 2 H), 4.11–4.21 (m, 1 H), 3.83 (s, 3 H), 3.56–3.63 (m, 1 H), 2.99–3.08 (m, 1 H), 2.76–2.85 (m, 1 H), 2.23–2.31 (m, 1 H), 2.18 (s, 3 H), 2.01–2.13 (m, 1 H), 1.85–2.00 (m, 1 H), 1.62–1.75 (m, 1 H). LCMS (ES$^+$, *m/z*): 614.3 [(M+H)$^+$].

Compound 29: N-(2,3-dihydroxypropyl)-4-[3-[4-[[(3S,4R)-3-fluoro-1-methyl-4-piperidyl]amino]-1-(2,2,2-trifluoroethyl)indol-2-yl]prop-2-ynylamino]-3-methoxy-benzamide

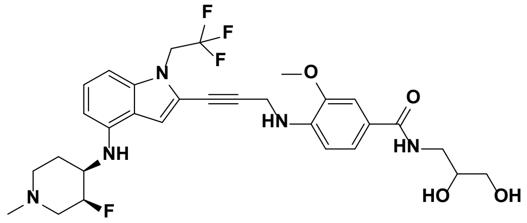

^{1}H NMR (400 MHz, DMSO-d_6) δ 8.17 (s, 1 H), 8.11–8.16 (m, 1 H), 7.45 (br d, J = 7.7 Hz, 1 H), 7.38 (br s, 1 H), 7.16 (s, 1 H), 7.00 (br t, J = 7.9 Hz, 1 H), 6.64–6.80 (m, 2 H), 6.23 (br d, J = 7.2 Hz, 1 H), 5.97–6.08 (m, 1 H), 5.48 (br d, J = 8.4 Hz, 1 H), 4.67–5.00 (m, 4 H), 4.58 (br dd, J = 2.5, 4.1 Hz, 1 H), 4.32 (br d, J = 5.4 Hz, 2 H), 3.85 (s, 3 H), 3.55–3.66 (m, 2 H), 3.48–3.52 (m, 1 H), 3.18 (br d, J = 6.7 Hz, 1 H), 3.02–3.09 (m, 1 H), 2.76–2.85 (m, 1 H), 2.24–2.32 (m, 1 H), 2.18 (br s, 3 H), 2.05–2.12 (m, 1 H), 1.83–1.95 (m, 1 H), 1.60–1.76 (m, 1 H). LCMS (ES$^+$, *m/z*): 606.3 [(M+H)$^+$].

Compound 30: [4-[3-[4-[[(3S,4R)-3-fluoro-1-methyl-4-piperidyl]amino]-1-(2,2,2-trifluoroethyl)indol-2-yl]prop-2-ynyl-amino]-3-methoxy-phenyl]-(4-methylpiperazin-1-yl)methanone

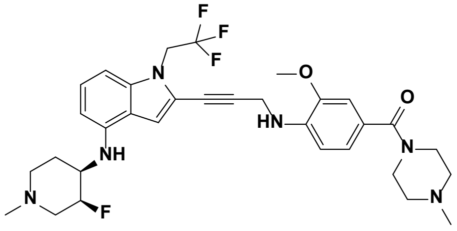

^{1}H NMR (400 MHz, DMSO-d_6) δ ppm 8.15 (s, 1 H), 7.17 (s, 1 H), 7.01 (t, J = 8.0 Hz, 1 H), 6.84–6.95 (m, 2 H), 6.71–6.80 (m, 2 H), 6.24 (d, J = 7.8 Hz, 1 H), 5.93 (t, J = 6.3 Hz, 1 H), 5.50 (br d, J = 8.6 Hz, 1 H), 4.72–5.00 (m, 3 H), 4.30 (br d, J = 6.1 Hz, 2 H), 3.81 (s, 3 H), 3.45–3.67 (m, 5 H), 3.05 (br t, J = 10.6 Hz, 1 H), 2.83 (br d, J = 10.9 Hz, 1 H), 2.28–2.34 (m, 4 H), 2.20 (d, J = 4.2 Hz, 6 H), 2.12 (br t, J = 10.9 Hz, 1 H), 1.93 (br dd, J = 3.4, 12.3 Hz, 1 H), 1.70 (br d, J = 9.9 Hz, 1 H). LCMS (ES$^+$, *m/z*): 615.4 [$(M+H)^+$].

Compound 31: 4-[3-[4-[[(3S,4R)-3-fluoro-1-methyl-4-piperidyl]amino]-1-(2,2,2-trifluoroethyl)indol-2-yl]prop-2-ynyl-amino]-3-methoxy-N-(1-methyl-4-piperidyl)benzamide

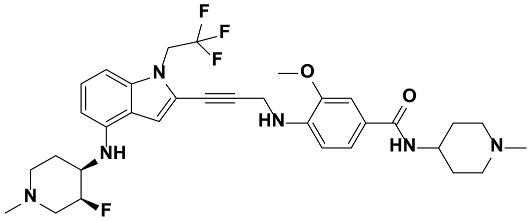

^{1}H NMR (400 MHz, DMSO-d_6) δ ppm 8.19 (s, 2 H), 7.93 (d, J = 7.7 Hz, 1 H), 7.46 (dd, J = 1.7, 8.3 Hz, 1 H), 7.35 (d, J = 1.7 Hz, 1 H), 7.16 (s, 1 H), 7.00 (t, J = 8.0 Hz, 1 H), 6.69–6.83 (m, 2 H), 6.24 (d, J = 7.8 Hz, 1 H), 5.99 (t, J = 6.2 Hz, 1 H), 5.48 (br d, J = 7.1 Hz, 1 H), 4.70–4.99 (m, 3 H), 4.28–4.36 (m, 2 H), 3.74–3.88 (m, 4 H), 3.50–3.63 (m, 1 H), 3.00–3.09 (m, 1 H), 2.95 (br d, J = 11.6 Hz, 2 H), 2.81 (br d, J = 10.9 Hz, 1 H), 2.24–2.35 (m, 5 H), 2.17–2.23 (m, 4 H), 2.11 (br t, J = 10.9 Hz, 1 H), 1.92 (br dd, J = 3.5, 12.1 Hz, 1 H), 1.80 (br d, J = 10.1 Hz, 2 H), 1.58–1.73 (m, 3 H). LCMS (ES+, *m/z*): 629.3 [$(M+H)^+$].

X-ray Crystallography Study

Crystals of the p53 Y220C mutant protein (amino acids 94–312, stabilized by four additional mutations M133L, V203A, N239Y, N268D) in 25 mM Na_2HPO_4, pH 7.2, 150 mM KCl, 5 mM DTT were grown in 0.02 M K_2HPO_4, 20% (v/v) glycerol, 14% (w/v) PEG-8000 and directly cryo-protected in the well solution supplemented with 1 mM of compound **9**. The dataset was collected at the Canadian Light Source beamline 08-ID, and the structure was solved at 1.70 Å resolution by molecular replacement with 2VUK using Phenix. Space group: *P 21 21 21* with two molecules per ASU. PDB code: 9BR4

Time-Resolved Fluorescence Resonance Energy Transfer (TR-FRET) Assay

DNA binding activity was measured by the TR-FRET assay. In this assay, the recombinant His_6-tag Y220C p53 DNA binding domain (DBD) protein binds to biotin labeled consensus DNA. The binding of recombinant His6-tag Y220C p53 DBD protein and biotin-labeled consensus DNA is detected by the fluorescence resonance energy transfer (FRET) between allophycocyanin (APC) conjugated anti-His_6 tag antibody and Europium-(Eu) conjugated Streptavidin. Test compounds, at 2 mM stock in DMSO, were diluted three-fold in serial dilutions in DMSO and 1.2 µL per well were added into 384-well polypropylene black plates (NUNC). Subsequently, 30 µL per well of 181 nM recombinant His_6-tag Y220C p53 DBD protein (amino acids #94-312) and 9.1 nM of APC conjugated anti-His_6 tag antibody (Columbia Biosciences) in ice-cold Assay Buffer 1 (50 mM Tris-HCl, pH 7.4, 75 mM KCl, 0.75 mM DTT and 0.2 mg/mL bovine serum albumin [BSA]) was added. As a background control, 30 µL of Assay Buffer 1 containing 9.1 nM of APC anti-His_6 antibody (no Y220C protein) was also added into a second set of serial diluted compound plates. The samples were centrifuged at 1,200 rpm (Eppendorf 5810R plate centrifuge) for 1 minute and incubated at room temperature (20 °C) for 15 min. The samples were then incubated at 27 °C in a water bath for 48 min. Afterwards, 5 µL per well of 145 nM biotin labeled oligonucleotide from a consensus DNA p53 response element (a DNA duplex with 5' to 3'sequence of ATTAGGCATGTCTAGGCATGTCTAGG and biotin attached to the 3' end of the complementary DNA) and 8.8 nM Eu-conjugated streptavidin (Columbia Biosciences) in Assay Buffer 2 (50 mM Tris-HCl, pH 7.4, 75 mM KCl and 0.2 mg/mL BSA) were added. The samples were centrifuged at 1,200 rpm for 1 min and incubated at room temperature (20 °C) for 20 min. Assay signals were monitored by reading excitation at 340 nm, and emission fluorescence at 615 and 665 nm, on an Envision reader (Perkin Elmer). Normalized TR-FRET assay signal (R_n) was calculated by the formula:

$$R_n = [(A - B_a - C \times (D - B_d))/(D - B_d)] \times (D_c - B_d),$$

Where A is the fluorescence intensity of the sample at 665 nm, D is the fluorescence intensity of the sample at 615 nm, B_a and B_d are plate backgrounds at 665 and 615 nm, respectively, and D_c is the fluorescence intensity of 0.67 nM Eu-conjugated streptavidin in the Assay Buffer at 615 nm.

The crosstalk factor (C) is determined by the following formula:

$C = (A_c - B_a)/(D_c - B_d)$,

Where A_c is the fluorescence intensity of 0.67 nM Eu-conjugated streptavidin in the Assay Buffer at 665 nm. The SC_{150} (substrate concentration to increase DNA binding by 1.5) value was calculated using either Prism (GraphPad) or XLfit software (IDBS).

Cell Proliferation Assay

The antiproliferative activity of compounds was evaluated using the 3-[4,5-dimethylthiazol-2-yl]-2,5 diphenyl tetrazolium bromide (MTT) assay in a 96-well plate format. Cells were seeded at an appropriate density depending on the growth rate in 96-well microtiter plates. Compounds were tested at eight concentrations with 2-fold serial dilutions. After 5 days, the antiproliferative activity was assessed by the conversion of the MTT dye (1 mg/ml) into insoluble formazan by viable cells. This formazan was dissolved in ethanol and measured using a microplate reader at an absorbance wavelength of 570 nm, with a reference wavelength of 650 nm. The results were presented as a percentage of the viability of untreated cells (control). The half-maximal inhibitory concentration (IC_{50}) was determined from the regression of a plot of the logarithm of concentration versus percent viability using XLfit IDBS.

Pharmacokinetic Study in Mice

Pharmacokinetic parameters were evaluated in female CD-1 mice. Animals received an oral gavage dose of 50 mg/kg of the test article. The test article was formulated in a solution containing 2% hydroxypropyl cellulose and 0.5% Tween 80 in water. Blood samples were collected at 0.5, 1, 2, 4, 7, and 24 h post dose. Blood samples were processed into plasma, and the concentrations of the test article were determined by LC-MS/MS. Pharmacokinetic parameters were calculated using the Phoenix WinNonlin 6.3 program, employing a non-compartmental model 200.

Study of Compound 26 in NUGC-3 Xenograft

Female Balb/c nude mice, aged 4 to 6 weeks, were obtained from Envigo (Madison, WI). All mice were housed at the PMV Pharmaceuticals Vivarium. Mouse care and experimental procedures adhered to institutional guidelines for ethical use, with all research protocols approved by the Institutional Animal Care and Use Committees of PMV Pharmaceuticals. The mice were maintained at ambient temperatures of 68–72 °F, with 30–70% humidity, and a 12-hour dark/light cycle.

NUGC-3 cells were resuspended at a concentration of 1×10^7 viable cells/mL in a 50:50 mixture of phosphate-buffered saline and Matrigel Matrix (Corning). Subsequently, 100 μL of the suspension was injected subcutaneously into the dorsal flank of the mice. Mice were selected and randomized into treatment groups based on tumor size.
